# Rank-based genome-wide analysis reveals the association of Ryanodine receptor-2 gene variants with childhood asthma among human populations

**DOI:** 10.1186/1479-7364-7-16

**Published:** 2013-07-05

**Authors:** Lili Ding, Tilahun Abebe, Joseph Beyene, Russell A Wilke, Arnon Goldberg, Jessica G Woo, Lisa J Martin, Marc E Rothenberg, Marepalli Rao, Gurjit K Khurana Hershey, Ranajit Chakraborty, Tesfaye B Mersha

**Affiliations:** 1 Department of Pediatrics, Cincinnati Children’s Hospital Medical Center, University of Cincinnati, Cincinnati, OH 45229, USA; 2 Department of Biology, University of Northern Iowa, Cedar Falls, IA 50614, USA; 3 Department of Clinical Epidemiology and Biostatistics, Program in Population Genomics, McMaster University, 1280 Main Street West, MDCL 3211, Hamilton, Ontario, L8S 4K1, Canada; 4 Department of Medicine, Division of Clinical Pharmacology, Oates Institute for Experimental Therapeutics, Vanderbilt University Medical Center, Nashville, TN 37232, USA; 5 Sapir Medical Center, Sackler Faculty of Medicine, Tel-Aviv University, Tel-Aviv 6997801, Israel; 6 Division of Epidemiology and Biostatistics, Department of Environmental Health, University of Cincinnati, Cincinnati, OH 45229, USA; 7 Department of Forensic and Investigative Genetics, Center for Computational Genomics, Institute of Applied Genetics, University of North Texas Health Science Center, Fort Worth, TX 76107, USA

**Keywords:** Asthma, GWAS, Ancestry, Trans-ancestral analysis, Rank analysis, Imputation, dbGaP, 1000 Genomes project, Networks/pathways, *RYR2*

## Abstract

**Background:**

The standard approach to determine unique or shared genetic factors across populations is to identify risk alleles in one population and investigate replication in others. However, since populations differ in DNA sequence information, allele frequencies, effect sizes, and linkage disequilibrium patterns, SNP association using a uniform stringent threshold on *p* values may not be reproducible across populations. Here, we developed rank-based methods to investigate shared or population-specific loci and pathways for childhood asthma across individuals of diverse ancestry. We performed genome-wide association studies on 859,790 SNPs genotyped in 527 affected offspring trios of European, African, and Hispanic ancestry using publically available asthma database in the Genotypes and Phenotypes database.

**Results:**

Rank-based analyses showed that there are shared genetic factors for asthma across populations, more at the gene and pathway levels than at the SNP level. Although the top 1,000 SNPs were not shared, 11 genes (*RYR2*, *PDE4D*, *CSMD1*, *CDH13*, *ROBO2*, *RBFOX1*, *PTPRD*, *NPAS3*, *PDE1C*, *SEMA5A*, and *CTNNA2*) mapped by these SNPs were shared across populations. Ryanodine receptor 2 (*RYR2*, a statin response-related gene) showed the strongest association in European (*p* value = 2.55 × 10^−7^) and was replicated in African (2.57 × 10^−4^) and Hispanic (1.18 × 10^−3^) Americans. Imputation analyses based on the 1000 Genomes Project uncovered additional *RYR2* variants associated with asthma. Network and functional ontology analyses revealed that *RYR2* is an integral part of dermatological or allergic disorder biological networks, specifically in the functional classes involving inflammatory, eosinophilic, and respiratory diseases.

**Conclusion:**

Our rank-based genome-wide analysis revealed for the first time an association of *RYR2* variants with asthma and replicated previously discovered *PDE4D* asthma gene across human populations. The replication of top-ranked asthma genes across populations suggests that such loci are less likely to be false positives and could indicate true associations. Variants that are associated with asthma across populations could be used to identify individuals who are at high risk for asthma regardless of genetic ancestry.

## Background

Asthma [MIM 600807] is a disease of chronic airway inflammation that affects over 300 million individuals worldwide, including 24.6 million in the USA [1]. It is estimated that asthma-related health care costs the US economy US$56 billion a year [2]. Asthma has important racial disparities in prevalence, morbidity, mortality, and drug response. In the USA, the prevalence of asthma varies between racial groups, ranging from 7.8% in European-Americans to 11.1% in African-Americans and up to 16.6% in Hispanic-Americans [3]. While differences in lifestyle and socioeconomic status between racial groups may contribute to differences in asthma prevalence, population genetic variation may be partly responsible for the current disparities in asthma susceptibility.

As of December 4, 2012, there are 28 genome-wide association studies (GWAS) that identified 78 SNP-asthma associations. The main strength of GWAS is its ability to systematically explore truly novel candidate SNPs/genes associated with chronic diseases. However, many SNPs identified by GWAS explain only a small fraction of the genetic risk [4-6]. Furthermore, there is selection bias toward ‘top hits’ in GWAS. As reported by Baye et al. [7], the problem of focusing on few top-hit SNPs is that if the *p* value threshold is set too low, genes that have little effect individually but are relevant to complex traits when they interact with other genes are not detectable. Recently, Torgerson et al. [8] conducted genetic association studies across asthmatic populations with a cutoff *p* value of 10^−6^ and discovered 34 SNPs in European-Americans, 4 SNPs in African-Americans and African-Caribbeans, 32 in the Hispanic-Americans, and 75 in the combined meta-analysis. Although such study can discover markers with large effect sizes, stringent cutoff values may not be realistic for across-population comparison given that each population has a unique genetic and demographic history and that populations vary in DNA sequence information, allele frequencies, effect sizes as well as exhibit heterogeneity in linkage disequilibrium (LD) patterns between the identified variants and the causative functional variants that underlie disease risk [9-12].

Studies based on gene sets (a) have a larger effect size on complex trait than individual SNPs, (b) have a greater power to detect functionally relevant genes, and (c) improve the interpretability and reproducibility of genetic studies on complex diseases [13]. Approaches that include genetic signals at all levels, for example, loci/gene and pathways, without an arbitrary threshold of statistical significance are needed. Such methods are capable of extracting more information from GWAS data by identifying loci that have functional similarities. We hypothesized that such an approach could generate sound biological bases for subsequent studies compared with studies that rely on single markers with low *p* values.

Currently, across-population studies on asthma genetics are limited and several questions are not properly addressed, including the following: How often are the same sets of SNPs, genes, or pathways associated with asthma across populations? To what degree are asthmatic subjects of different populations enriched for common sets of susceptible loci? Answering these questions systematically will allow us to understand risk variants for asthma that are population-specific or shared across populations and implement better interventions for asthma. If a common set of loci are associated with asthma across populations, then it is reasonable to hypothesize that those loci are more likely to share one or more pathways compared to loci that are not shared or associated with asthma. With the availability of data generated on the same commercial SNP chips (i.e., high level overlap in the SNP sets), we have the opportunity to compare genome-wide associations with asthma across populations directly and sift the wheat from the chaff [14]. Therefore, the objective of the current study was to systematically analyze the presence of shared or population-specific genetic risk factors for asthma among European, African-American, and Hispanic asthmatic children at the locus and pathway levels. To accomplish this, we performed genome-wide association analysis of childhood asthma using 859,790 SNP markers genotyped in a sample of 527 affected offspring trios of different racial groups. Affected offspring trio or family design is robust against population substructure, which is of particular concern when studying African-Americans or Hispanic-Americans with diverse ancestry contributions. Comparing with case–control studies, where cases and controls are often unrelated, affected offspring trio design avoids population and DNA quality differences between the cases and controls and the possibility that some controls borrowed from other studies might be affected with the phenotype of interest.

## Results

### Description of study subjects and association

Table 1 shows racial distribution and the number of SNPs genotyped for each population, shared by all three populations, and specific to each population in the Childhood Asthma Research and Education (CARE) and Childhood Asthma Management Program (CAMP) databases. There are 859,790 autosomal SNPs genotyped in the three populations that passed inclusion criteria; among them 786,195 SNPs (91.4%) are shared by all three populations. The number of population-specific SNPs that are polymorphic in only one of the three populations is 688 for European-American, 3,705 for African-American, and 180 for Hispanic-American population.

**Table 1:** The number of affected offspring trios and number of SNPs by population

	**Number of affected offspring trios**			**Number of SNPs**	
	**CAMP**	**CARE**	**CAMP and CARE**	**Genotyped**	**Population-specific**^**a**^
Population					
EA	334	95	429	842,915	688
AA	42	10	52	855,949	3,705
HA	30	16	46	846,188	180
Total	406	121	527	859,790^b^	4,573
Shared				786,195^c^	

*EA*, European-American; *AA*, African-American; *HA*, Hispanic-American populations. ^a^Number of SNPs that passed inclusion criteria and polymorphic in only one of the three populations. ^b^Number of SNPs that passed inclusion criteria and polymorphic in at least one of the three populations. ^c^Number of SNPs that passed inclusion criteria and polymorphic in all three populations.

### Population-specific associations

**Figure 1:**
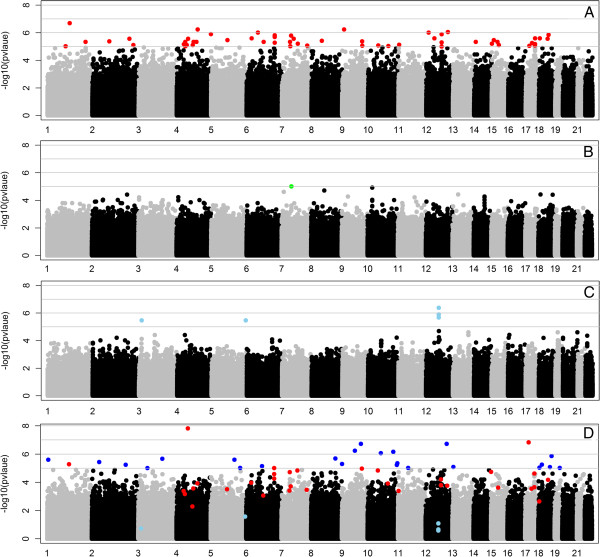
**Manhattan plots of the trans-ancestral analysis. (A)** European-American, **(B)** African-American, **(C)** Hispanic-American population, and **(D)** all three populations combined. The *y*-axis displays the negative logarithm of the *p* value for each SNP marker; the *x*-axis displays the markers’ genomic coordinates by chromosome. In **(A)**, **(B)**, and **(C)**, colored dots (red for European-Americans, green for African-Americans, and light blue for Hispanic-Americans) indicate markers with *p* value <1 × 10^−5^. Among these markers, those that passed inclusion criteria in the mega-sample are also indicated with their respective colors in **(D)**, where the additional markers with *p* value <1 × 10^−5^ in the mega-analysis are indicated in blue.

Figure 1 shows the Manhattan plots of transmission disequilibrium test (TDT) results for each population separately and all three populations combined (mega-analysis). More significant results were obtained for European-American than African or Hispanic ancestry populations. Although we see little genomic inflation factors (λ) (ranging from 1.03 to 1.2) in this study, our ranked-based approach was not influenced by inflation, and global genomic correction factor is not relevant. In other words, the order of the markers based on rank analysis did not change with correction for the genomic inflation factor (data not shown). Additional file 1: Tables S1, S2, S3, and S4 show the top 100 SNPs of each population and the mega-analysis of the combined data. The *p* values of the top 100 SNPs ranged from 1.81 × 10^−7^ to 7.74 × 10^−6^ for European-American, 9.76 × 10^−6^ to 2.72 × 10^−4^ for African-American, 4.20 × 10^−7^ to 2.57 × 10^−4^ for Hispanic-American population, and 8.33 × 10^−9^ to 9.58 × 10^−6^ for the mega-analysis. The top 100 SNPs were not shared between any two or among all three populations. Among the top 100 SNPs of the combined data, 18, 1, and none are shared in the top 100 SNPs of European-American, African-American, and Hispanic-American population, respectively. From mega-analysis, we showed that simply combining diverse data may not result in the identification of variants important in all populations.

Among the genes that mapped to the top 100 loci of European-Americans, *CNTN1* (Contactin 1, [MIM 600016]) was proposed to have an important function in the invasion and metastasis of lung adenocarcinoma cells [15]. The gene *STAT5A* (Signal transducer and activator of transcription 5A, [MIM 601511]) was indicated to be critical in STAT6-independent Th2 cell differentiation and allergic airway inflammation [16]. Among the genes mapped from the top 100 loci of African-Americans, Ryanodine receptor-2 (*RYR2* [MIM 180902]) has been implicated in the calcium response that leads to increased airway contraction [17,18] and extensive airway narrowing, which characterizes a key event underlying asthma. Two other top genes in African-Americans (*CDH13* [MIM 601364] and *PTPRD* [MIM 601598]) are related to lung cancer and childhood asthma [19-21]. Among the top genes in Hispanic-American population, *RBFOX1* (RNA binding protein, fox-1 homolog, *C. elegans*, 1 [MIM 605104]) was reported to be related to survival in lung cancer patients [22]. *DAPK1* (Death-associated protein kinase 1, [MIM 600831]) was shown to be associated with cell death and inflammatory and immunological diseases. *DOCK1* (Dedicator of cytokinesis 1 [MIM 601403]) was reported to be moderately associated with asthma [23].

Additional file 1: Table S5 shows the number of SNPs with *p* values below the cutoffs (0.05, 0.01, 10^−4^, 10^−5^, and 10^−6^) in each population, in the combined sample, and shared between populations. The three populations shared 180 and 2 SNPs at a *p* value cutoff of 0.05 and 0.01, respectively. No SNPs were shared by any two or all three of the populations with more stringent *p* value cutoffs (<10^−4^), which may be partly due to the genetic heterogeneity across the study populations and the small sample size of the study populations. Thus, instead of *p* value cutoff, we focused on top-ranked SNPs/genes and pathways/gene ontologies (GOs) in the rest of our analyses.

### Little overlapping among top-ranked SNPs

The left side of Table 2 shows the number of SNP markers shared by any two populations or all three populations among the top-ranked SNPs. Among the top 1,000 SNPs, 2 were shared by European-Americans and African-Americans, 2 by European-Americans and Hispanic-Americans, and none by African-Americans and Hispanic-Americans. For the sets of top 2,000 SNPs, European-Americans and African-Americans shared 4 SNPs, European-Americans and Hispanic-Americans shared 7 SNPs, and African-Americans and Hispanic-Americans shared none. When the top 10,000 SNPs were considered, three SNPs were shared by all three populations, 2 more than what would be expected by chance alone. Table 3 shows these SNPs and their *p* values in each population. Among these shared sites, *ARSB* (Arylsulfatase B, [MIM 611542]) was shown to regulate colonic epithelial cell migration [24].

**Table 2:** SNP level and gene level overlap

**Number of top SNPs**	**SNP level**				**Gene level**						
	**Overlap**				**Mapped genes**			**Overlap**^a^			
	**EA and AA**	**EA and HA**	**AA and HA**	**EA, AA, and HA**	**EA**	**AA**	**HA**	**EA and AA**	**EA and HA**	**AA and HA**	**EA, AA, and HA**
1,000	2	2	0	0	328	299	252	41/5	30/4	29/4	11/0
2,000	4	7	0	0	616	531	450	110/16	77/12	72/14	34/0
5,000	39	40	33	0	1,272	1,121	961	322/71	266/54	242/61	126/3
10,000	170	235	145	3	2,120	1,882	1,677	686/199	624/158	575/178	353/18
50,000	3,549	4,228	3,463	284	5,600	5,515	5,193	3,163/1,544	3,041/1,431	2,962/1,454	2,151/401

*EA*, European-American; *AA*, African-American; *HA*, Hispanic-American populations. ^a^Observed/expected by chance.

**Table 3:** Shared SNPs among top-ranked SNPs

**rs ID**	**Gene**	**Chr**	***p *value**		
			**EA**	**AA**	**HA**
Shared SNPs among top 2,000 SNPs of EA and AA					
rs7045156		9	3.74 × 10^−5^	2.50 × 10^−3^	3.17 × 10^−1^
rs1048329	*LRP2BP*	4	8.89 × 10^−4^	1.86 × 10^−3^	1
*rs12359404*	*SORCS1*	10	4.07 × 10^−4^	5.32 × 10^−4^	3.17 × 10^−1^
*rs16875946*	*ARSB*	5	4.65 × 10^−4^	1.70 × 10^−3^	4.55 × 10^−2^
Shared SNPs among top 2,000 SNPs of EA and HA					
*rs2928442*		10	1.39 × 10^−4^	1.17 × 10^−1^	1.62 × 10^−3^
*rs2272266*	*PLA1A*	3	1.23 × 10^−4^	6.55 × 10^−1^	1.57 × 10^−3^
rs4128918		5	6.40 × 10^−4^	NA	2.70 × 10^−3^
rs4008848		9	8.72 × 10^−4^	7.15 × 10^−1^	3.50 × 10^−3^
rs9913559	*RDM1*	17	2.06 × 10^−4^	8.19 × 10^−1^	2.84 × 10^−3^
rs6550392		3	9.11 × 10^−4^	3.53 × 10^−1^	2.70 × 10^−3^
rs9301462	*RAB20*	13	7.10 × 10^−6^	8.82 × 10^−1^	2.70 × 10^−3^
Shared SNPs among top 10,000 SNPs of EA, AA, and HA					
rs920672	*NAV2*	11	2.67 × 10^−5^	1.43 × 10^−2^	1.43 × 10^−2^
rs11021111		11	5.56 × 10^−4^	1.16 × 10^−2^	1.26 × 10^−2^
rs1314595	*ATRNL1*	10	3.12 × 10^−4^	1.16 × 10^−2^	8.15 × 10^−3^

SNPs in italics were among the top 1,000. *EA*, European-American; *AA*, African-American; *HA*, Hispanic-American populations; *Chr*, chromosome.

Although the same loci were not shared among the top-ranked SNPs across populations, many top-ranked SNPs of one population were replicated in the other two populations at a nominal *p* value of 0.05. Table 4 shows among the top 100 SNPs in each population how many had *p* value less than 0.05 in the other two populations or the mega-analysis, where the expectation by chance alone is 5. Among the top 100 SNPs of European-American population, 8 in African-American and 18 in Hispanic-American population had *p* value < 0.05. Among the top 100 SNPs of African-American, 7 in European-American and 2 in Hispanic-American population had *p* value < 0.05. Among the top 100 SNPs of Hispanic-American population, 10 in European-Americans and 5 in African-Americans had *p* value <0.05. Additional file 1: Tables S1, S2, S3, and S4 show these SNPs and their *p* values in all three populations and in the mega-analysis. For example, the most significant SNP in European-Americans (rs16929097, *p* value = 1.81 × 10^−7^) was replicated in African-Americans with a *p* value of 0.0114, and the second most significant SNP in European-Americans (rs17036023, *IGSF3* [MIM 603491], *p* value = 2.04 × 10^−7^) was replicated in Hispanic-American population with a *p* value of 0.0143. One of the top 100 SNPs of European-Americans (rs16863100, *p* value = 7.10 × 10^−6^) was replicated in both African-Americans (*p* value = 0.0412) and Hispanic-Americans (*p* value = 0.0143). One of the top 100 SNPs in Hispanic-Americans (rs13486, *p* value = 8.77 × 10^−5^) was replicated in both European-Americans (*p* value = 0.0365) and African-Americans (*p* value = 0.0039).

**Table 4:** Replication of top 100 SNPs of each population in other populations

**Population**	**Number of *p *values <0.05**			
	**EA**	**AA**	**HA**	**Mega**
EA	100	8	18	81
AA	7	100	2	33
HA	10	5	100	15
Mega	100	43	42	100

Replication is defined at a nominal *p* value of 0.05. EA, European-American; AA, African-American; HA, Hispanic-American populations; Mega, mega-analysis based on combined samples from all three populations.

### Replication of top-ranked asthma genes

Among the top 1,000 SNPs, 403, 417, and 405 SNPs were mapped to 328, 299, and 252 genes, and interquartile ranges (IQR) of the numbers of SNPs mapped to each gene were (1, 1), (1, 1), and (1, 2) for European-American, African-American, and Hispanic-American population, respectively. Among the mapped genes, European-American and African-American populations shared 41 genes, European-American and Hispanic populations shared 30 genes, and African-American and Hispanic populations shared 29 genes (Table 2). In addition, there are 11 genes shared by all the three populations. These 11 genes are *RYR2*, *CSMD1* [MIM 608397], *CDH13*, *ROBO2* [MIM 602431], *RBFOX1* [MIM 605104], *PTPRD*, *NPAS3* [MIM 609430], *PDE1C* [MIM 602987], *SEMA5A* [MIM 609297], *CTNNA2* [MIM 114025], and *PDE4D* [MIM 600129]. Table 5 lists the *p* values of these genes in the three populations, which ranged from 2.55 × 10^−7^ to 1.62 × 10^−3^. *RYR2* is a statin response-related gene that showed the strongest association in European-Americans (*p* value = 2.55 × 10^−7^) and was replicated in African-Americans (2.57 × 10^−4^) and Hispanic-Americans (1.18 × 10^−3^). *PDE4D* was identified as an asthma susceptibility gene, and PDE4 inhibitors have been developed as medications for asthma [25]. Variants in *PTPRD* were reported to be associated with childhood asthma in Taiwanese population [20].

**Table 5:** Eleven genes shared by the top 1,000 SNPs of each population

**Genes**	**Chr**	***p *values**		
		**EA**	**AA**	**HA**
*RYR2*	1	2.55 × 10^−7^	2.57 × 10^−4^	1.18 × 10^−3^
*CSMD1*	8	4.23 × 10^−6^	4.15 × 10^−4^	4.18 × 10^−4^
*CDH13*	16	2.01 × 10^−5^	2.31 × 10^−4^	1.60 × 10^−3^
*ROBO2*	3	2.01 × 10^−5^	3.93 × 10^−4^	1.36 × 10^−3^
*RBFOX1*	16	2.21 × 10^−5^	1.76 × 10^−3^	3.86 × 10^−5^
*PTPRD*	9	2.38 × 10^−5^	2.08 × 10^−4^	3.12 × 10^−4^
*NPAS3*	14	3.74 × 10^−5^	1.58 × 10^−3^	1.02 × 10^−3^
*PDE1C*	7	9.62 × 10^−5^	9.41 × 10^−4^	1.32 × 10^−3^
*SEMA5A*	5	1.08 × 10^−4^	4.03 × 10^−4^	1.34 × 10^−3^
*CTNNA2*	2	1.83 × 10^−4^	1.28 × 10^−3^	1.57 × 10^−3^
*PDE4D*	5	1.83 × 10^−4^	4.02 × 10^−4^	1.62 × 10^−3^

*EA*, European-American; *AA*, African-American; *HA*, Hispanic-American populations; *Chr*, chromosome.

**Figure 2:**
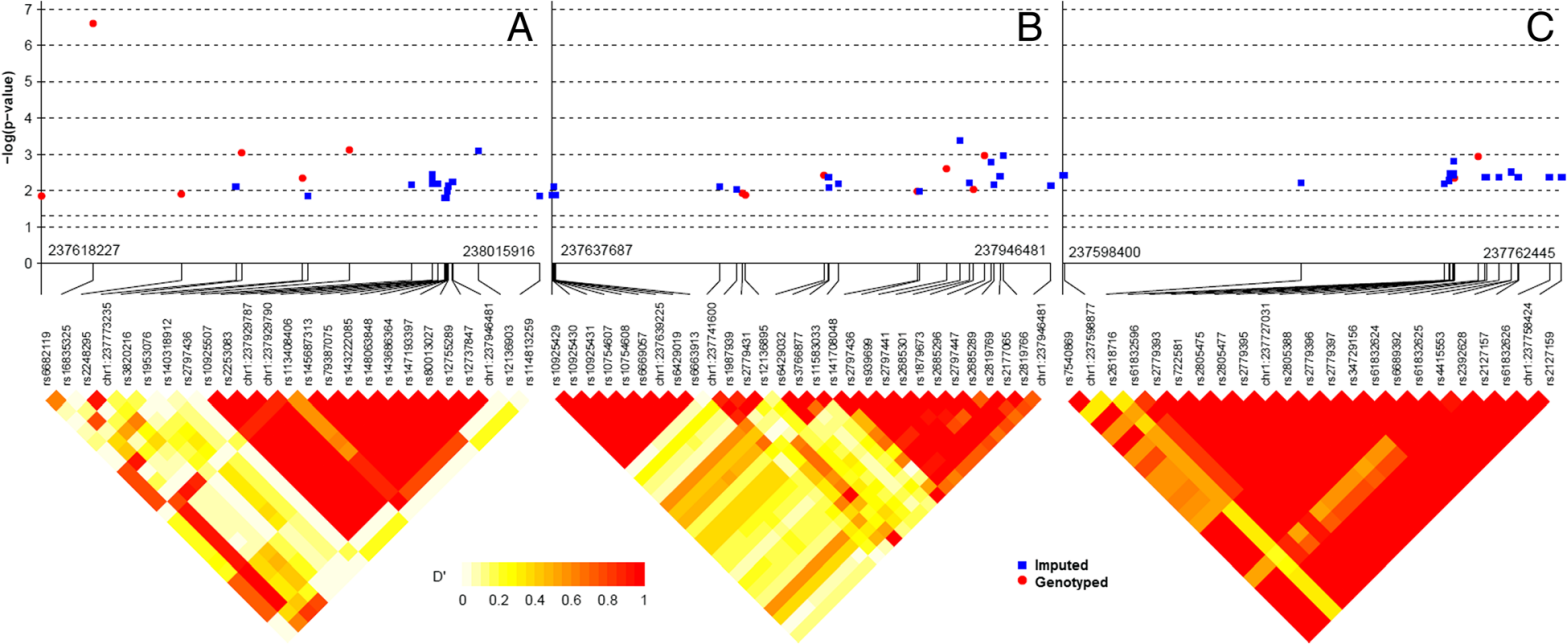
**The *p *value and LD plot of post-imputation RYR2 SNPs for each population.** Trans-ancestral analysis of genotyped and imputed association results and LD of the top 1% SNPs of *RYR2* after imputation. **(A)** European-American population, **(B)** African-American population, and **(C)** Hispanic-American population.

To detect additional variants in the top-ranked *RYR2* asthma gene across populations, we imputed untyped SNPs in *RYR2* using haplotypes from the 1000 Genomes Project as reference panels. The number of SNPs in this gene that passed the filtering criteria in European-American, African-American, and Hispanic-American populations was 262, 382, and 371, respectively, before imputation, and 2,533, 2,884, and 2,304 after imputation. Post-imputation SNPs were then ranked from most significant to least significant according to their association with asthma. Figure 2 shows the *p* value and LD plot of the top 1% of the post-imputation SNPs for each population. Additional file 1: Table S6 lists these SNPs, their *p* values in each population, and possible functional effects. In European-Americans, no imputed SNPs exceeded the strongest association inferred from the genotyped SNP (rs16835325, 2.55 × 10^−7^). One imputed SNP (rs12136903) showed moderate association with asthma (*p* value = 7.89 × 10^−4^). In African-Americans, the strongest signal from genotyped SNPs (rs2797447, *p* value = 1.09 × 10^−3^) was exceeded by an imputed SNP (rs2685301, *p* value = 4.15 × 10^−4^) that is within a LD block of four of the top-ranked genotyped SNPs. In Hispanic-Americans, association from imputed SNPs again supported signals from genotyped SNPs. Two imputed SNPs showed strong LD with genotyped SNPs and second to the best association with asthma (rs2779359 and chr1:237727031 both with *p* value = 1.60 × 10^−3^).

### Top-ranked pathways and GO terms

**Figure 3:**
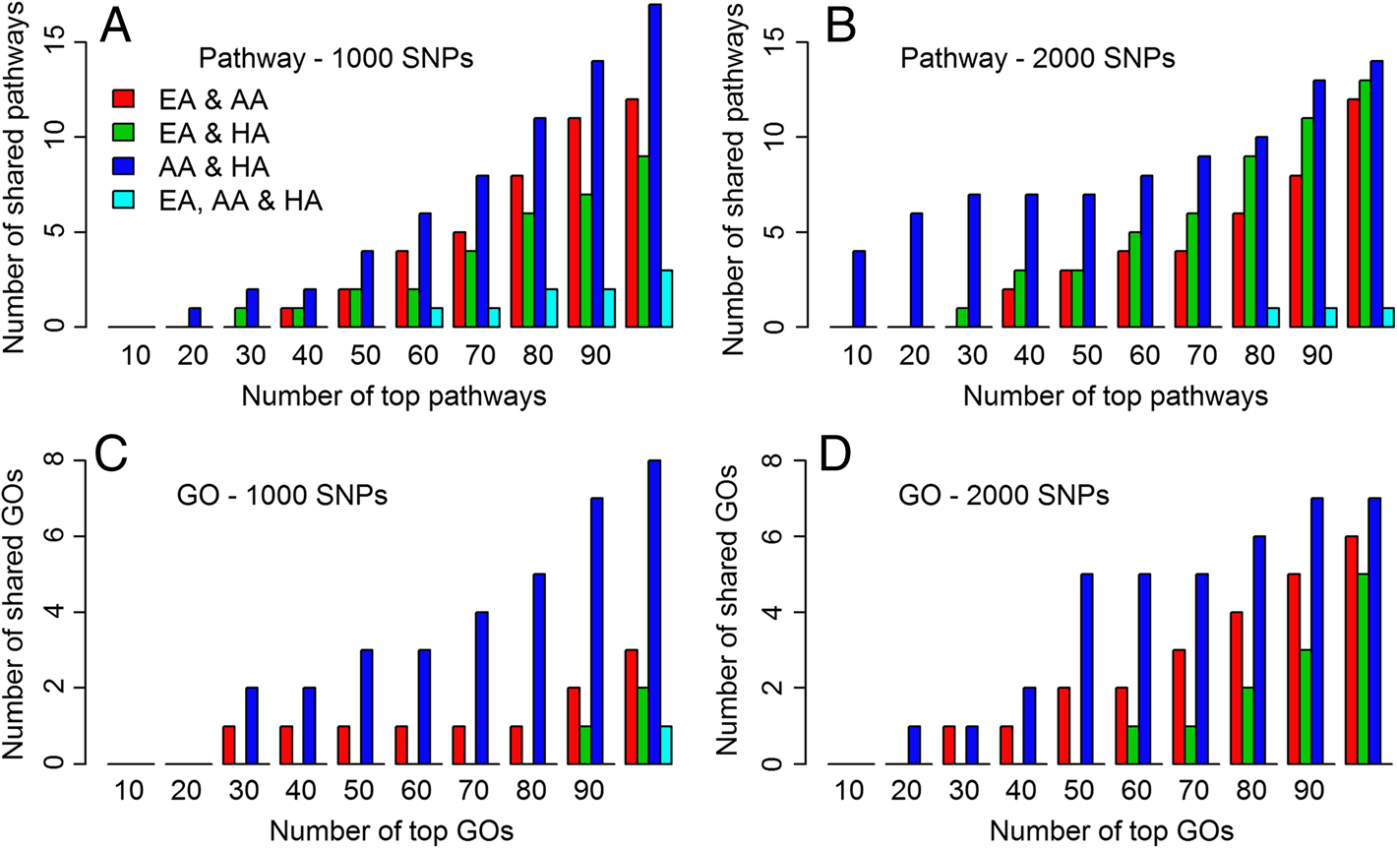
**Overlap of genetic risk factor for childhood asthma across the three populations: At the pathway and GO level.** The number of shared pathways (*y*-axis) among different numbers of top-ranked pathways (*x*-axis) when the top **(A)** 1,000 and **(B)** 2,000 SNPs were declared as noteworthy. The number of shared GO terms (*y*-axis) among different numbers of top-ranked GO terms (*x*-axis) when the top **(C)** 1,000 and **(D)** 2,000 SNPs were declared as noteworthy. EA, European-American; AA, African-American; HA, Hispanic-American populations.

To gain further insights into the pathogenesis of asthma and determine significant biological pathways and gene ontologies and to reveal genes associated with asthma across populations, we conducted gene set analysis based on pathways and GO terms. Table 6 shows the pathways and GOs that were over-represented with *p* value < 0.01 in at least one of the three populations when the top 1,000 SNPs were declared as noteworthy. Figure 3 shows the amount of overlap across the three populations at the pathway and GO level. When the top 1,000 SNPs were declared as noteworthy, African-American and Hispanic-American populations shared 1 of their top 20 pathways (Prefoldin mediated transfer of substrate to *CCT*/*TRiC*) and 1 of their top 30 GOs (ribonuclease activity); European-American and African-American populations shared 1 of their top 40 pathways (Shc-mediated cascade), and African-American and Hispanic-American populations shared 1 of their top 30 pathways (Stathmin pathway) and 2 of their top 30 GO terms (cellular macromolecule catabolic process and the macromolecule catabolic process). In addition, among the three sets of top 60 pathways, only 1 pathway was shared by all three populations (arrhythmogenic right ventricular cardiomyopathy). Additional file 1: Table S7 and S8 show the top 30 pathways and GOs for each population and their *p* values when the top 1,000 SNPs were declared as noteworthy. When less stringent top 2,000 SNPs were considered, African-American and Hispanic populations shared four of their top ten pathways (systemic lupus erythematosus, packaging of telomere ends, RNA polymerase I promoter clearance, and RNA polymerase I promoter opening). Two more pathways were shared by the two populations among their top 20 pathways (RNA polymerase I and III and mitochondrial transcription and telomere maintenance). In particular, we found enrichment of the leukocyte trafficking pathway which indicates that the accumulation and activation of inflammatory leukocytes in the lung or airway is a feature shared by almost all respiratory diseases [26]. The leukocyte trafficking pathway has been suggested to have a key role in asthma, which makes the finding in our study biologically plausible. Children with asthma may be considered to suffer from chronic inflammatory stress [27].

**Table 6:** Over-represented pathways and GOs

	***p *values**		
	**EA**	**AA**	**HA**
GO			
Cellular response to stress	0.0006	1	1
Vitamin binding	0.0056	0.3392	1
JAK STAT cascade	0.0062	1	0.4304
Vitamin transport	0.0072	0.3210	0.2886
ER nuclear signaling pathway	0.0078	1	1
Regulation of gene-specific transcription	0.0078	1	1
Positive regulation of cell proliferation	0.4326	0.0006	0.4412
Lysosomal transport	1	0.0016	1
Carbohydrate binding	0.6714	0.0038	0.8342
RNA catabolic process	0.3710	0.0050	0.2818
Vacuolar transport	1	0.0052	1
Negative regulation of catalytic activity	0.7328	0.1720	0.0004
Protein kinase binding	1	0.7450	0.0016
Kinase binding	0.8654	0.5054	0.0040
Regulation of translation	0.8478	1	0.0050
Anion transmembrane transporter activity	1	0.7148	0.0080
Negative regulation of hydrolase activity	1	1	0.0100
Pathway			
BioCarta G2 pathway	0.0008	1	1
Reactome E2F transcriptional targets at G1 S	0.0034	1	1
Reactome E2F mediated regulation of DNA replication	0.0036	1	1
KEGG alanine aspartate and glutamate metabolism	0.5218	0.0032	0.4338
BioCarta longevity pathway	0.4426	0.0088	1
Reactome amine ligand binding receptors	0.3642	0.1202	0.0006
Reactome G alpha S signaling events	0.3682	0.8264	0.0060

With *p* value <0.01 in at least one of the three populations when the top 1,000 SNPs are declared as noteworthy. *EA*, European-American; *AA*, African-American; *HA*, Hispanic-American populations; *KEGG*, Kyoto encyclopedia of genes and genomes.

### Higher population genetic differentiation was found at variants that show association with asthma

Table 7 shows summary statistics of fixation index (*F*_ST_) and the relationship between population genetic differentiation and population differences in association with asthma for the shared SNPs among populations. Compared with the *F*_ST_ values between European-American and Hispanic-American populations, those between African-Americans and European-Americans (or Hispanic-Americans) were higher, suggesting more population genetic differentiation between African-Americans and European-Americans (or Hispanic-Americans). Furthermore, population genetic differentiation was stronger in asthmatic individuals (affected offspring) than the non-asthmatic individuals (parents). Regardless of the data used (affected offspring or parents), the observed numbers of markers that satisfy difference between the rankings of *p* values (DRP) > mean of DRP and *F*_ST_ > mean of *F*_ST_ were higher than the expected number of markers. The dependence between population genetic differentiation and population differences in association with asthma was significant for European-American vs. African-American population and for European-American vs. Hispanic-American population. However, no significant relationship existed for African-American vs. Hispanic-American population. We believe this is due to either the relatively similar asthma prevalence in African-Americans and Hispanic-Americans or different mechanisms of asthma association in European-American and African/Hispanic-American populations. We conclude that for populations with different asthma prevalence, such as European-Americans vs. African-Americans and European-Americans vs. Hispanic-Americans, SNPs that are more informative for ancestry or exhibit large population genetic differentiation are more likely to be different in their association with asthma in the different populations.

**Table 7:** Population genetic differentiation and population differences in genetic association with asthma

**Allele frequencies in CAMP and CARE**	***F***_**ST**_			**DRP > mean (DRP) and *F***_**ST**_ **> mean (*F***_**ST**_**)**		
	**Mean**	**Median**	**Range**	**Observed**	**Expected**	***p *values**^**a**^
From parents						
Caucasian vs. African-American	0.0463	0.0263	(0, 0.5858)	108,245	107,720	0.007898
Caucasian vs. Hispanic	0.0078	0.0036	(0, 0.1770)	95,757	94,447	7.749 × 10^−12^
African-American vs. Hispanic	0.0387	0.0215	(0, 0.4989)	107,740	107,384	0.07227
From the affected offspring						
Caucasian vs. African-American	0.0487	0.0274	(0, 0.6036)	107,987	107,432	0.004965
Caucasian vs. Hispanic	0.0114	0.0050	(0, 0.2226)	98,522	97,445	2.39 × 10^−8^
African-American vs. Hispanic	0.0433	0.0238	(0, 0.5429)	107,458	107,225	0.2408

^a^*p* values of the chi-square test of the frequency table of DRP > mean (DRP) and *F*_ST_ > mean (*F*_ST_). DRP, difference between the rankings of *p* values in the two populations.

### Patterns of variation across populations

**Figure 4:**
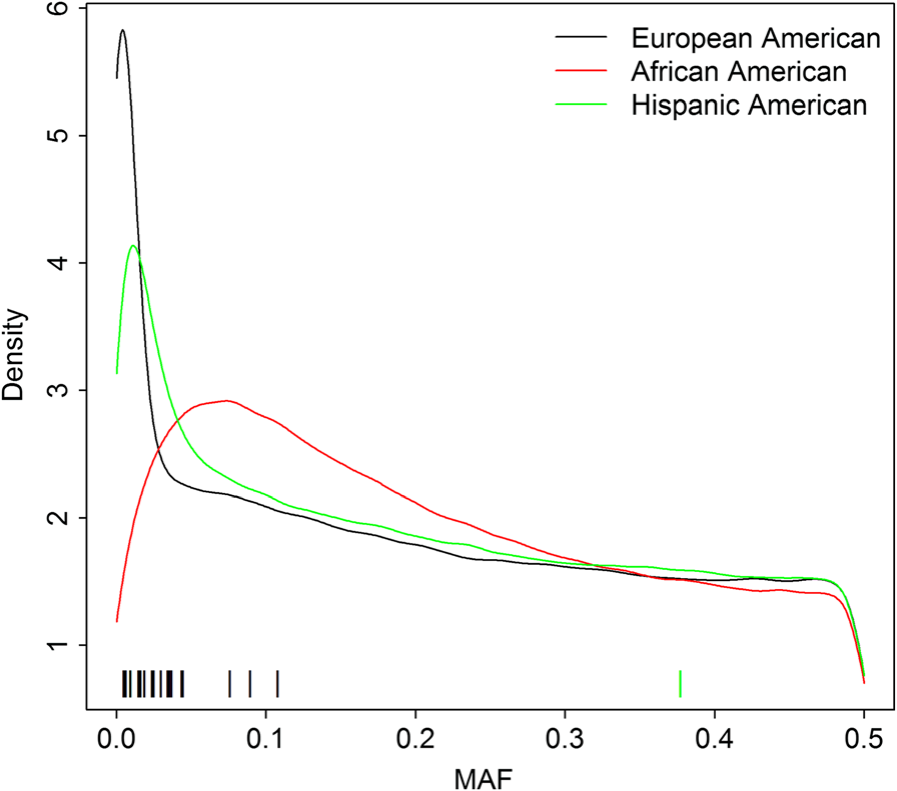
**Density plot of MAF.** The MAFs were estimated using the affected offspring. The bars above the *x*-axis indicate the MAF of SNPs with *p* value <1 × 10^−6^ in each population.

**Figure 5:**
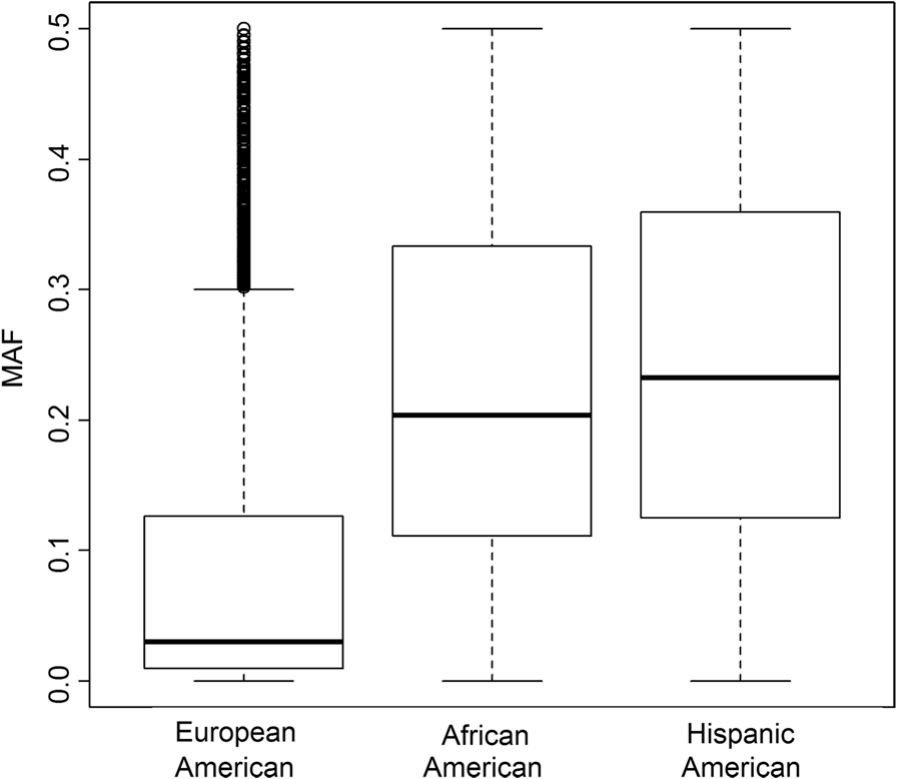
**Box and whisker plots of MAF of the top 5,000 SNPs for each population.** The MAFs were estimated using affected offspring. The bottom and top of the box are the lower and upper quartiles, respectively, the band within the box is the median, and the ends of the whiskers are the lowest/highest data value within 1.5 IQR of the lower/higher quartile. IQR is the difference between the upper and the lower quartiles.

Figure 4 shows the density plot of minor allele frequencies (MAF) of the three populations estimated using affected offspring. For European-Americans, MAF had mean = 0.20, median = 0.19, and IQR = (0.06, 0.34); for African-American subjects, MAF mean = 0.22, median = 0.19, and IQR = (0.09, 0.33); and for Hispanic-American subjects, the MAF mean = 0.21, median = 0.19, and IQR = (0.07, 0.34). Overall, MAFs in European-Americans were smaller than African-American and Hispanic-American populations and had a relatively larger peak at the lower end of the MAF spectrum, which may be due to the current reference genomes and commercial SNP panels included in the Affymetrix 6.0 genotyping chip being primarily selected based on identification and patterns of LD in European ancestry population. This might also indicate that the European-American population is less heterogeneous and less diverse compared with African-Americans and Hispanic-Americans since African-American and Hispanic-American individuals are of admixed origin, while the European-Americans are simply of varied European ancestry. Figure 5 shows the box and whisker plot of MAF of the top 5,000 SNPs for each population. Similar pattern was observed for the top 1,000 or 2,000 SNPs. MAFs of top-ranked SNPs in European-Americans (mean = 0.09, median = 0.03, IQR = (0.01, 0.13)) were on average much lower than those in African-Americans (mean = 0.23, median = 0.20, IQR = (0.11, 0.33)) and Hispanic-Americans (mean = 0.24, median = 0.23, IQR = (0.13, 0.36)). Compared to overall MAF, top-ranked SNPs had lower MAF in European-Americans. Recent studies show that variants altering amino acid sequence and protein function are enriched at low variant allele frequency, 2% to 5% [28].

## Discussion

Several studies have explored shared genetics among diseases including coeliac disease and other immune diseases [29], non-Hodgkin's lymphoma and autoimmune diseases [30], obesity and asthma [21], and asthma and chronic obstructive pulmonary disease [31]. However, there is very little investigation into population-specific or shared genetic risk factors for a specific disease across different populations. In this report, we described the results of GWAS asthma associations in three populations, namely European-Americans, African-Americans, and Hispanic-Americans. The method we used is based on the ranking of SNP associations from the most to the least significant and testing in the context of functionally relevant genes and gene networks. We observed that there are shared genetic risk factors (genes and pathways) for asthma across populations, although none of the top-ranked SNPs associated in each population was replicated in others. The heterogeneity of top GWAS ‘hits’ could be a result of a combination of ancestry variations in the study populations, differences in asthma phenotype definitions and unaccounted-for environmental factors.

When the top 10,000 SNPs for each population were considered, only 3 SNP were found to be shared by all three populations (rs1314595, *ATRNL1* [MIM 612869], chromosome (chr) 10, with *p* value = 0.0003 in European-Americans, 0.0082 in Hispanic-Americans, and 0.0116 in African-Americans; rs920672, chr 11, with *p* value = 2.67 × 10^−5^ in European-Americans and 0.0143 in both African- and Hispanic-Americans; and rs11021111, chr 11, with *p* value = 0.0006 in European-Americans, 0.0126 in Hispanic-Americans, and 0.0116 in African-Americans). As suggested by Jansen et al*.*[32], whenever information from multiple independent sources agree, it is more likely the findings are valid and reliable than information from a single source. Hence, replication of top-ranked asthma genes or pathways across data from different populations is a way to validate population-specific findings, and such associations are less likely to be false positives and could indicate functionality. In fact evolutionary geneticists used the idea that ‘genes that are conserved across populations are likely to be functionally important, since they would confer a selective advantage to all humans’ [33].

Among the top 1,000 SNPs of each population, there were 11 loci shared by all three populations, and the genes encoded by these loci are *PDE4D*, *RYR2*, *CSMD1*, *CDH13*, *ROBO2*, *RBFOX1*, *PTPRD*, *NPAS3*, *PDE1C*, *SEMA5A*, and *CTNNA2* (*p* values ranged from 2.55 × 10^−7^ to 1.62 × 10^−3^, Table 5). *PDE4D* (phosphodiesterase 4D) functions as a regulator of airway smooth muscle contractility and was identified as an asthma susceptibility gene, and PDE4 inhibitors have been developed as medications for asthma [25]. Variants in *PTPRD* (protein encoded by protein tyrosine phosphatase receptor-type delta) gene were reported to be associated with childhood asthma in Taiwanese population [20]. Melen et al. [21] in their study of shared genetic factors between asthma and obesity in children found association between *PTPRD* with both phenotypes at the gene level (*p* < 0.05). *NPAS3* (neuronal PAS domain protein 3) encodes a member of the basic helix-loop-helix and PAS domain-containing family of transcription factors. Zhou et al. [34] showed that *NPAS3* haploinsufficient mice survived postnatally but developed alveolar loss and airway hyperreactivity. Genome-wide linkage has identified linkage peak at chromosome 14q12-13 region, where *NPAS3* maps in asthmatic Caucasians [35-37]. *ROBO2* belongs to the Roundabout (ROBO) family, part of the immunoglobulin superfamily proteins that are highly conserved from fly to human. The encoded protein is a receptor for and essential for signal transduction of Slit2, a secreted protein that is known to function in axon guidance and cell migration, plays a critical role in the development of normal airways [38], and is an important etiologic factor in airway narrowing that accompanies asthma [39].

The *RYR2* gene is located from base pair 237,205,701 to base pair 237,997,287 on chromosome 1. Mutations in *RYR2* are causative of dysfunctional calcium channel which often results in sudden cardiac death [40]. Recent genome-wide association studies have also associated *RYR2* variants with muscle toxicity and a potential pharmacodynamic candidate gene in statin response-related disorders; *RYR2* encodes a ryanodine receptor and contributes to the calcium response that leads to increased airway contraction and extensive airway narrowing, which characterizes a key event underlying asthma [17,18,41]. In a recent GWAS study, an intronic variant (rs2819742) in *RYR2* was significantly associated with cerivastatin-associated rhabdomyolysis at the *a priori p* value threshold of 4 × 10^−7^ (*p* = 1.74 × 10^−7^). An additional copy of the minor allele of the *RYR2* variant was associated with a reduced risk of rhabdomyolysis (odds ratio (OR) = 0.48; 95% confidence interval (CI) = 0.36 to 0.63). Carriers of two copies of the minor allele had a smaller risk of rhabdomyolysis than carriers of two copies of the major allele (OR = 0.24; 95% CI = 0.13 to 0.44) [42]. As the associated SNPs in the *RYR2* gene were common variants and could be due to linkage disequilibrium from untyped functional variants, we imputed both rare and common variants using the 1000 Genomes Project reference panel. Imputation can also permit the comparison of studies which focused on different SNPs. Using genotypes inferred through imput-ation, we uncovered additional *RYR2* variants (rs2685301 in African-Americans and rs2779359 in Hispanic-Americans) that exhibited moderate association with asthma and significant LD with genotyped SNPs.

**Figure 6:**
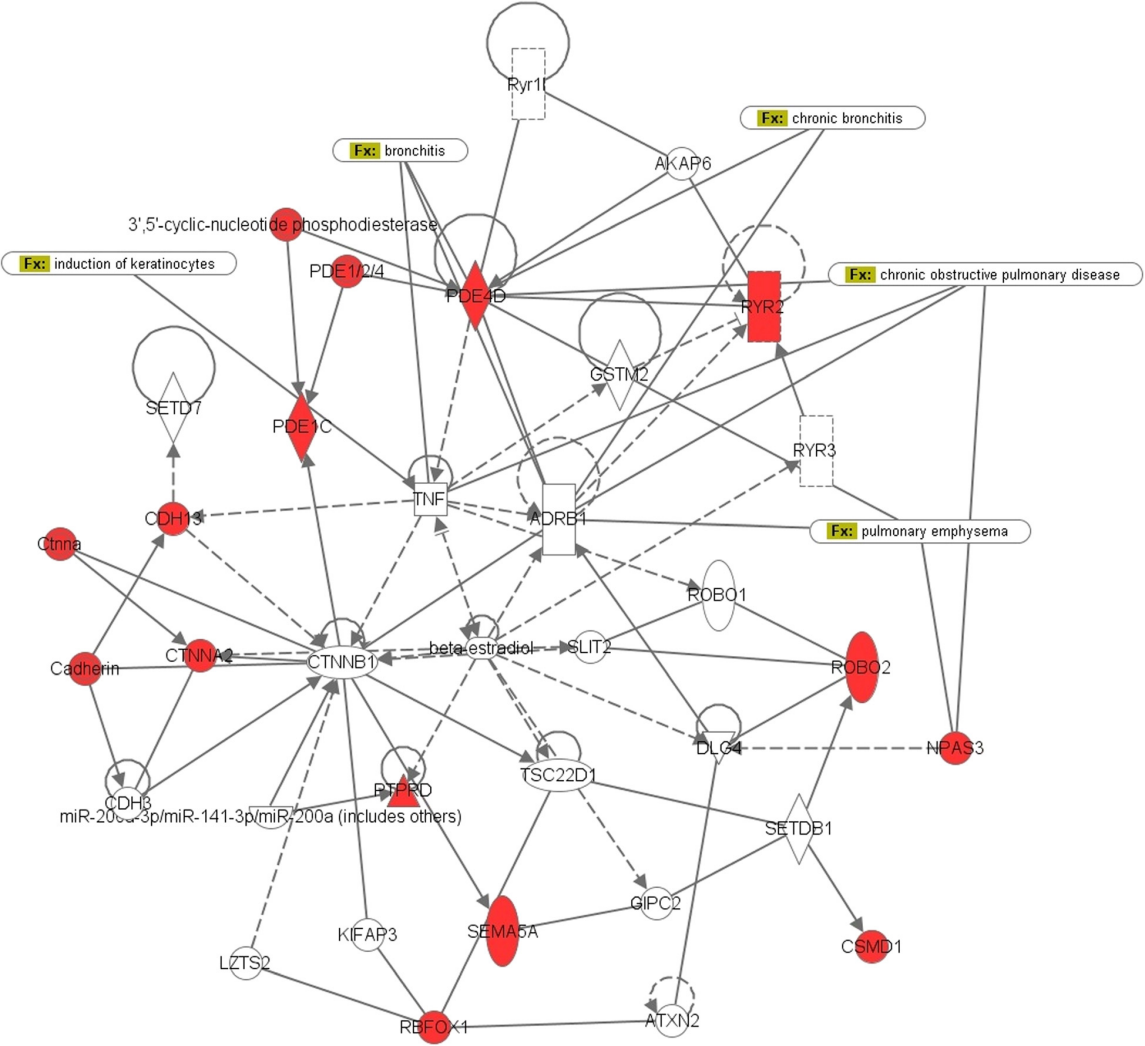
**Interactive network of the 11 genes shared among the top 1,000 SNPs of each population.** The 11 genes are *CDH13*, *CSMD1*, *CTNNA2*, *NPAS3*, *PDE1C*, *PDE4D*, *PTPRD*, *RBFOX1*, *ROBO2*, *RYR2*, and *SEMA5A*. Genes with red nodes represent hub genes in our analysis; others are generated through the network analysis from Ingenuity Pathways Knowledge Base. Edges are displayed with labels that describe the nature of the relationship between the nodes. All edges are supported by at least one reference from the literature or from canonical information stored in the Ingenuity Pathways Knowledge Base. The lines between genes represent known interactions, with solid lines representing direct interactions and dashed lines representing indirect interactions. Nodes are displayed using various shapes that represent the functional class of the gene product.

Complementary to rank-based candidate gene selection for a given disease, gene network analysis offers the advantage of understanding the interaction of functionally related genes that are associated with a disease and the ability to find hub genes within a network that interact with several other genes up- and downstream of them. The high interconnectivity of hub genes with other correlated genes within a biological network may imply functional and biological importance of these genes. Further analysis using Ingenuity Pathways Analysis (IPA) revealed that the 11 genes shared among top-ranked loci of the three populations are part of integrated and interconnected biological networks related to dermatological or allergic disorders, particularly in the functional classes involving inflammatory and immunological diseases (Figure 6). This could reflect that the development of asthma involves the ‘atopic march’ that starts in the skin and progresses to the respiratory and gastrointestinal tracts [43]. At the center of the network is the ‘hub’ *CTNNB1* (Catenin (cadherin-associated protein), beta 1, [MIM 116806]) gene complex [44,45], which occurs at cell-cell junctions in epithelial tissues and constitutes adherens junctions. Many studies showed the importance of β-catenin as signaling pathway in airway smooth muscle growth [46]. The role for E-cadherin in asthma has also been studied. Heijink [47] suggested that E-cadherin controls the response to allergens, suppresses allergenic mediator production, and contributes to the establishment of tolerance. De Boer et al. [48] showed that the expression of epithelial alpha-catenin and E-cadherin is lower in atopic asthma patients, and this may result in a defective epithelial barrier in the airway epithelium, which plays a critical role in asthma. Table 8 lists the top diseases and disorders related to these genes where inflammatory and respiratory diseases are among them. Shared pathways across all three populations were observed among the top 60 pathways from the top 1,000 SNPs, and top 100 GO terms from the top 1,000 SNPs (Figure 3).

**Table 8:** Top diseases and disorders related to the 11 genes shared among the top 1,000 SNPs across populations

** Functions**	***p *values**	**Genes**
Cardiovascular disease	1.90 × 10^−5^–3.86 × 10^−2^	*CDH13*, *CSMD1*, *PDE4D*, *RBFOX1*, *RYR2*
Genetic disorder	1.90 × 10^−5^–4.21 × 10^−2^	*CDH13*, *CSMD1*, *PDE4D*, *RBFOX1*, *RYR2*, *ROBO2*, *NPAS3*, *SEMA5A*
Hematological disease	1.90 × 10^−5^–1.63 × 10^−2^	*CDH13*, *CSMD1*, *PDE4D*, *RBFOX1*, *RYR2*
Psychological disorders	7.15 × 10^−4^–3.45 × 10^−2^	*NPAS3*, *RBFOX1*, *SEMA5A*, *PDE4D*
Inflammatory disease	1.16 × 10^−3^–4.21 × 10^−2^	*NPAS3*, *PDE4D*, *RYR2*
Respiratory disease	1.16 × 10^−3^–2.05 × 10^−2^	*NPAS3*, *PDE4D*

Although we are limited by our modest sample size in this study, it is important to note that the CAMP and CARE affected offspring trio design have extremely well characterized subjects with detailed phenotypic data. It should be noted that large sample sizes may not help in powering genetic studies and improve our understanding on the genetic underpinnings of asthma phenotypes as much as precise phenotyping [49]. Further, the trio design is robust against population substructure, which is of particular concern when studying African-Americans or Hispanic-Americans with diverse ancestry in case–control study design, where cases and controls are defined variably. Although there are different methods (such as genomic control, structured association, and principle component analysis) to correct for confounding, a good study design is the most efficient way to avoid confounding in disease genetics study. The use of family-based designs increases the power to detect associations, controls for heterogeneity/population stratification, and might elucidate the effects of allele origin as well as transmission of phenotypes of disease modulation. The order of markers based on our rank-based approach remains the same before and after correcting for genomic inflation factor (an indication of the scale-invariant nature of the ranking method). Genetic heterogeneity among ethnic groups, which has been a source of concern in GWAS, will not affect pathway-based GWAS analysis. This is because although the mutated genes or variants within those pathways are likely to differ, affected individuals from different ethnic groups may share the same disrupted pathways. Thus, multiple GWAS can be easily combined, and pathway-based GWAS accommodate and capitalize upon this substantial degree of genetic heterogeneity. The current reference genomes and commercial tagging SNP panels included in the Affymetrix 6.0 genotyping chip were primarily selected based on higher minor allele frequencies and patterns of LD in European ancestry population. Thus, due to relatively weaker LD and variation in minor allele frequencies, for example, in African populations, we may not have the power to detect all of the genetic variants involved in asthma in this population as demonstrated by the 1000 Genomes Project imputation analysis. Indeed, whole-genome sequencing may be necessary to identify population-specific variants in less studied populations such as African-Americans and Hispanics. Eventually, next-generation sequencing technologies will overtake SNP arrays as the primary and less biased genotyping methodology and advance our understanding on rare variants and population-specific influences on disease risk. Additional functional analysis is also necessary to more fully understand the roles that ancestry-specific variants at these loci play in asthma.

## Conclusions

In summary, our rank-based approach avoids the need for a global cutoff value for statistically significant associations. Importantly, since we did not rely on a statistical cutoff to classify significant SNPs, our approach is not susceptible to biases due to SNP density or LD structure [50]. This approach is more appropriate to compare disease association results across populations that vary in DNA sequence, allele frequencies, effect sizes, linkage disequilibrium patterns, and gene-by-environment interactions. We showed the existence of shared genetic risk factors for childhood asthma across the European-American, African-American, and Hispanic-American populations. Our rank-based genome-wide analysis revealed for the first time an association of *RYR2* variants with asthma and replicated previously discovered *PDE4D* asthma gene across human populations. The shared association of asthma for a given gene across populations might likely indicate true association and a broader spectrum of risk factor at the loci. The associations of particular variants (or even different variants within the same gene) across populations and across studies may represent more universally important genes to the disorder and should be given the highest priority [51]. Often, these variants may not be the strongest associations in any one study, but the consistent evidence for association in many different studies (e.g., as revealed in our different racial groups study) would further suggest that the variant and gene have main effects on the phenotype, are less likely influenced by gene-gene or gene-environment interactions, and are most likely to be true associations. Network analysis revealed that *RYR2* and *PDE4D* genes are directly interacting in biological networks. Regulating the expression of both genes along with the hub genes such as *CTNNB1* could be important in the treatment of asthma across populations. Hub genes tend to be conserved across evolution. Thus, hub genes represent towards the evolutionary fitness of an organism, and alterations in their sequence or expression level are likely to be more deleterious. In this study, our goal was to identify variants that consistently associated with asthma across populations and analytical methods. We believe that, once validated, such cross-population variants could be used to identify individuals who are at high risk for asthma regardless of genetic ancestry. Additional studies are necessary to further elucidate biological roles of *RYR2* and pathways related to *RYR2* genes in asthma pathogenesis.

## Methods

### Subjects

dbGaP data from the CAMP and the CARE Network were used to assess shared and population-specific risk variants for childhood asthma across three populations, namely European-American, African-American, and Hispanic-American. CAMP and CARE are part of the SNP Health Association Resource Asthma Resource project, which is a genome-wide analysis of children who have participated in the National Heart, Lung, and Blood Institute’s clinical research trials on asthma. dbGaP was developed to archive and distribute the results of studies that have investigated the interaction of genotype and phenotype. Such studies include genome-wide association studies, medical sequencing, molecular diagnostic assays, as well as association between genotype and non-clinical traits (http://www.ncbi.nlm.nih.gov/gap). This database provides consistently well-defined phenotypes measured across population.

**Figure 7:**
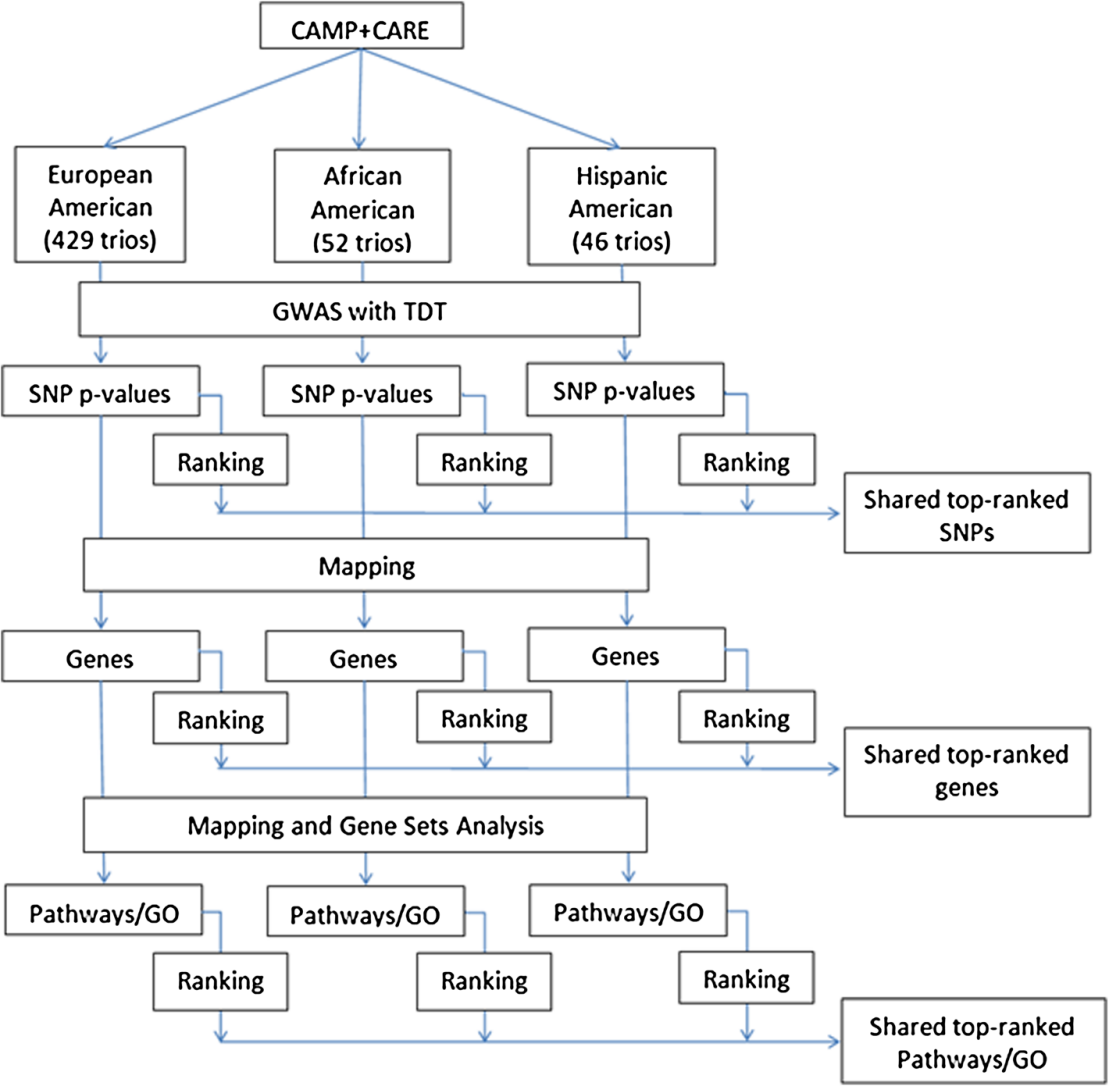
**Work flow diagram.** For each population, single-SNP analysis was first conducted using the family-based TDT. SNPs were then mapped to genes, and genes were mapped to pathways/gene sets based on annotation databases. Pathway level associations with childhood asthma were obtained based on gene set analysis. Overlapping and population-specific top-ranked genetic risk factors across the three populations at the locus and pathway levels were studied to investigate shared or unique pathophysiological processes in the study population.

We downloaded genotyping data performed using 1 million SNPs in the Affymetrix 6.0 chip and stored in the database of dbGaP with permission under the accession number phs000166.v2.p1. In the three populations, a total of 859,790 autosomal SNP markers passed the quality control filtering criteria (less than 15% missing data and Hardy-Weinberg equilibrium *p* values >10^−6^) and were included in the association analysis. Our approach was as follows: for each population, single-SNP analysis was first conducted using the family-based TDT. SNPs were then mapped to genes, and genes were mapped to gene sets, e.g., pathways and/or GO. Pathway level associations with childhood asthma were obtained based on gene set analysis. Loci and pathways were then ranked based on the *p* value of association in the order of the most significant to the least significant. Overlapping and population-specific top-ranked genetic risk factors across the three populations at the locus and pathway levels were studied to investigate shared or unique pathophysiological processes in the study population. Figure 7 shows the work flow diagram. Alternatively, to search for shared genetic risk factors for childhood asthma, a mega-analysis with combined subjects from the three populations was conducted since the TDT is valid in the presence of population structure.

The datasets used in this manuscript were obtained from previously collected, completely anonymized/deidentified, IRB-approved and NIH Controlled Access dbGaP data under accession number phs000166.v2.p1 at http://dbgap.ncbi.nlm.nih.gov.

### Statistical analysis

#### TDT for affected offspring trio design

The family-based TDT [52] is one of the most frequently used methods for family-based linkage/association studies. It evaluates the transmission frequency of an allele from heterozygous parents to affected offspring. Following the notations in Spielman et al. [52], among 2*q* parents of *q* affected offspring, Table 9 summarizes combinations of transmitted and non-transmitted alleles *M*_1_ and *M*_2_ at a bi-allelic locus *M*.

**Table 9:** **Combinations of transmitted and non-transmitted alleles *M***_**1 **_**and *M***_**2 **_**at a bi-allelic locus *M***

	**Non-transmitted allele**		**Total**
	***M***_**1**_	***M***_**2**_	
Transmitted allele			
*M*_1_	*a*	*b*	*a + b*
*M*_2_	*c*	*d*	*c + d*
Total	*a + c*	*b + d*	2*q*

The null hypothesis of no linkage between marker *M* and a disease susceptibility locus *D* can be expressed as *θ* = 0.5, where *θ* is the recombination fraction between *M* and *D*. Under the null hypothesis, the contributions from two heterozygous parents are independent. When only data from heterozygous parents (*M*_1_*M*_2_) are used, the TDT is a standard approximation of the binomial test of the equality of the two proportions: b/(b + c) and c/(b + c). The statistic is

$$\chi^{2}=\frac{{\left[b-\left(b+c\right)/2\right]}^{2}+{\left[c-\frac{b+c}{2}\right]}^{2}}{\frac{b+c}{2}}=\frac{{\left(b-c\right)}^{2}}{b+c}$$

Under the null hypothesis of no linkage or no association, the statistic has an asymptotic chi-square distribution with 1 degree of freedom. The TDT is based on preferential allelic transmissions at a SNP site from heterozygous parents to the affected offspring. The rejection of the null hypothesis implies the lack of recombination between the tested marker and the disease susceptibility locus. The TDT is robust against spurious associations due to population stratification.

#### Genetic association analysis based on rank

For each population, the TDT was carried out using PLINK v1.07 [53]. SNPs were then ranked based on the *p* value of association from the most significant to the least significant. A set of top *n* (1,000 to 50,000) SNPs were identified for each of the three populations. The top-ranked SNPs were then assigned to genes if the SNP is located within 20 kb of that gene. This is because the majority of trait-associated loci are located either within genes or no more than 20 kb outside the genes [54,55]. Shared loci between any two populations or among all three populations were quantified by examining overlapping among the three sets of top *n* SNPs and their mapped genes.

Two reasons prompted us to study the top-ranked loci instead of classifying statistically significant association using a threshold on *p* values. First, an optimal threshold is difficult to identify especially for multiple populations that differ in DNA sequence, allele frequencies, effect sizes, and LD patterns. Second, studying the same number of top loci from each population based on ranking of *p* values generates common results without confounding with sample size. We reason that if a high-ranking locus, although may not reach genome-wide significance, is shared or involves a common pathway across all three populations, it is more likely to have a causative disease association.

#### Imputation

For top-ranked asthma genes across populations, imputation of the untyped SNPs was performed using IMPUTE2 with settings recommended for imputation with an ancestrally diverse reference panel. Haplotypes from the 1000 Genomes Project [56] (Phase I integrated variant set release v3 in NCBI build 37) were used as multi-population reference panels [57]. Association analysis was then done on imputed SNPs using PLINK after the same method of filtering (i.e., less than 15% missing data and Hardy-Weinberg equilibrium *p* values >10^−6^). Association *p* values and linkage disequilibrium of top-ranked SNPs in the top-ranked gene were examined and plotted using snp.plotter [58].

#### Pathway/gene ontology analysis

Pathway analysis groups genes that are related biologically and tests whether these gene groups are associated with asthma. The goal is to detect association by integrating signals of multiple loci that are grouped into a pathway based on shared biological functions. Pathway analysis can also improve the interpretability and reproducibility of GWAS partly due to the substantial reduction of the multiple testing burden once genes are grouped into pathways [13]. Due to population genetic heterogeneity, different SNPs in or near the same gene or in a functionally related gene may be associated with the disease among individual cases in a GWAS sample. This makes it less likely that a replicable association with the disease would be found when testing SNPs one at a time as is usually done in a GWAS. Pathway-based tests provide a dynamic biologically plausible template to efficiently integrate statistical information from the multitude of SNPs with weaker effects that are otherwise missed by conventional single-SNP GWAS analysis. Statistical analyses of GWAS data that use biological pathways are represented by gene sets instead of SNPs, as the units of analysis are valuable. Gene set-based pathway analysis was first developed for gene expression studies and aimed to detect statistically significant changes in the expression of gene sets [59-63]. Recently, the method has been adapted for GWAS [55,64-67]. The first step of pathway-based analysis is the assignment of genes to gene sets based on existing annotation databases. We considered pathways and GO terms provided by the Molecular Signatures Database (MSigDB) v3 [63]. MSigDB includes 880 pathways that are canonical representations of a biological process. These pathways contain 186 KEGG [68] gene sets, 217 BioCarta gene sets, 430 Reactome gene sets [69], and 47 gene sets contributed by Signaling Gateway, Sigma Aldrich, Signaling Transduction KE, and SuperArray. MSigDB also provides 1,454 GO categories [70] which include 825 gene sets derived from biological processes (sets of molecular events with a defined beginning and end), 233 gene sets from cellular components (the parts of a cell or its extracellular environment), and 396 gene sets from molecular function categories (the activities of gene products at the molecular level).

The Association List GO Annotator algorithm, proposed by Holmans et al. [65] and implemented in the R package SNPath [71], was used to determine if a pathway/GO term is jointly associated with the trait of interest. The algorithm evaluates whether noteworthy genes are over-represented in a particular gene set compared with genes in the rest of the genome. Genes were declared noteworthy if the most significant SNP within the gene was noteworthy (e.g., among the top 1,000 SNPs of a particular population). A gene set test statistic was then computed based on a modified Fisher's exact test, and significance was finally assessed by gene re-sampling, which is much less computationally intensive compared to permutations of disease status or sample labels. For each population, a list of top-ranked pathways/GO was obtained. Commonality among the top-ranked pathways/GO across the three populations was then investigated.

#### Ingenuity pathways analysis

Pathway analyses on those 11 genes shared among top-ranked loci of the three populations that are associated with asthma were accomplished using Ingenuity Pathways Analysis 8.6 (Ingenuity Systems, Mountain View, CA, USA). The goal was to determine whether these genes in the three populations were part of integrated and interconnected biological networks of genes that have non-random enriched functional commonalities among the study subjects. A data set containing the eleven gene names was uploaded into IPA software to map and generate putative networks based on the manually curated knowledge database of pathways that was developed from a manual review of more than 200,000 scientific articles. The gene networks were generated using both direct and indirect relationships/connectivity. These networks were ranked by scores that measured the probability that the genes were included in the network not by chance alone.

#### Trans-ancestral analysis on combined samples

Following our population-based GWAS analysis, we also conducted a mega-analysis (by combining the data from the three populations) to improve the power to detect associated variants as a result of increased sample size. Only SNPs that passed the filtering criteria in all three populations were included in the mega-analysis. The association results from the mega-analysis are valid since the TDT results are not affected by population structure. Merging samples, however, does have potential drawbacks. Mixing populations could dilute association signals if recombination has separated a causal variant from a genotyped marker in some of the populations.

### Population genetic differentiation and association with asthma

Next, we analyzed levels of population genetic differentiation and investigated their relationship with population difference in asthma associations. The rationale is that if SNPs/genes that show population differentiation in both allele frequency and association with asthma exist, these SNPs/genes may partly explain the population differences in disease prevalence. A similar approach was used by Kovacic et al. (2011) [72], where the authors prioritized candidate SNPs/genes for childhood asthma by examining allelic frequency differences between populations with different asthma prevalence.

In our study, genetic differentiation between any two populations for a particular SNP was measured using fixation index, F_ST_, and population difference in association with asthma was measured by the difference between the rankings of the *p* values of the SNP in each population. We then examined the relationship between the difference in the rankings of *p* values and values of the F_ST_ measure. Frequency tables of DRP > mean of DRP and F_ST_ > mean of F_ST_ were constructed, and the independence between the two was tested using a chi-square test. The observed and expected numbers of SNPs with both DRP > mean of DRP and F_ST_ > mean of F_ST_ were compared. When the observed number is larger than the expected number, and the chi-square test gives a significant *p* value (< 0.05), we can conclude that SNPs that are more informative on ancestry or exhibit large population genetic differentiation are also more likely to be different in their disease association in different populations. These analyses were conducted for any two pair of the three populations, where the F_ST_ was calculated using either parents or the affected offspring in the CARE and CAMP data. We expect to see significant association between DRP and F_ST_ for populations with difference in asthma prevalence.

## Abbreviations

CAMP: Childhood asthma management program; CARE: Childhood asthma research and education; dbGaP: The database of genotypes and phenotypes; GO: Gene ontologies; GWAS: Genome-wide association studies; IPA: Ingenuity pathways analysis; LD: Linkage disequilibrium; MAF: Minor allele frequencies; MSigDB: Molecular signatures database; TDT: Transmission disequilibrium test.

## Competing interests

The authors declare that they have no competing interests.

## Authors’ contributions

TBM conceived of the study and drafted the manuscript with LD. LD performed the data analysis. TA, JB, RAW, AG, JGW, LJM, MER, MR, GKKH, and RC contributed to the manuscript writing. All authors read and approved the final manuscript.

## Supplementary Material

Additional file 1 **Supplemental data on the rank-based genome-wide analysis on Ryanodine receptor-2 gene variants in childhood asthma.** Contains **Tables S1**, **S2**, **S3**, **S4**, **S5**, **S6**, **S7**, and **S8**. Click here for file
