# Supplementary material for: Rank-based genome-wide analysis reveals the association of Ryanodine receptor-2 gene variants with childhood asthma among human populations
Source: Hum Genomics. 2013 Jul 5;7(1):16. doi: 10.1186/1479-7364-7-16 (PMC3708719; doi:10.1186/1479-7364-7-16)
Supplement: Additional file 1 — Supplemental data on the rank-based genome-wide analysis on Ryanodine receptor-2 gene variants in childhood asthma. Contains Tables S1, S2, S3, S4, S5, S6, S7, and S8. [file 1479-7364-7-16-S1.pdf]

Supplemental Data

Rank based genome wide analysis reveals association of ryanodine receptor-2 gene variants with childhood asthma among human populations

Lili Ding, Tilahun Abebe, Joseph Beyene, Russell A. Wilke, Arnon Goldberg, Jessica G. Woo, Lisa J. Martin, Marc E. Rothenberg, Marepalli Rao, Gurjit K. Khurana Hershey, Ranajit Chakraborty, Tesfaye B. Mersha

Table S1: Top 100 SNPs of European American population.

Table S2: Top 100 SNPs of African-American population.

Table S3: Top 100 SNPs of Hispanic population.

Table S4: Top 100 SNPs of trans-ancestral mega-analysis.

Table S5: Number of SNPs with p-value below cutoffs.

Table S6: top 1% SNPs of RYR2 after imputation for each population.

Table S7: Top 30 pathways of each population when top 1,000 SNPs are declared as noteworthy.

Table S8: Top 30 GO terms of each population when top 1K SNPs are declared as noteworthy.

Table S1: Top 100 SNPs of European American population. SNPs are ordered by p-values in the European American population (P1). When available, their p-values in African Americans (P2), Hispanic Americans (P3), and the mega-analysis (P123) are provided. P-values in bold are < 0.05.

rsID	Gene	Chr	BP	P1	P2	P3	P123
rs16929097		9	12521826	1.81E-07	**1.14E-02**	4.14E-01	**8.33E-09**
rs17036023	*IGSF3*	1	117129711	2.04E-07	6.95E-01	**1.43E-02**	**5.20E-06**
rs8091379		18	52491880	2.04E-07	2.21E-01	3.17E-01	**1.93E-03**
rs16835325	*RYR2*	1	237659306	2.55E-07	1.17E-01	7.63E-01	**6.58E-03**
rs12570188	*HPSE2*	10	100855702	3.19E-07	8.33E-02	1.57E-01	**4.60E-08**
rs16974314	*UNC13C*	15	54508722	3.41E-07	5.27E-01	8.33E-02	**1.54E-05**
rs1843978		8	89925447	3.46E-07	NA	2.57E-01	NA
rs11000019	*PSAP*	10	73591530	4.46E-07	3.17E-01	8.33E-02	**7.62E-08**
rs6054973		20	7385958	5.34E-07	1.66E-01	**4.55E-02**	**1.11E-07**
rs11217942	*GRIK4*	11	120533008	5.36E-07	NA	8.33E-02	NA
rs13435403		4	115235779	5.73E-07	8.66E-01	5.88E-02	**1.18E-04**
rs12353399	*C9ORF68*	9	4635448	5.73E-07	NA	1.80E-01	NA
rs431470		3	11945930	5.93E-07	NA	1.00E+00	NA
rs6836368		4	130751286	6.52E-07	7.89E-01	1.17E-01	**4.02E-05**
rs17479		5	5542927	6.91E-07	NA	7.15E-01	NA
rs2705520	*ATG3*	3	112269287	8.95E-07	5.64E-01	1.03E-01	**1.87E-06**
rs7971754	*CUX2*	12	111565969	8.95E-07	4.56E-01	3.17E-01	**1.74E-04**
rs17153000		8	11084501	9.23E-07	6.02E-01	1.03E-01	**1.10E-05**
rs17033506		3	35639826	9.63E-07	4.14E-01	8.33E-02	**4.46E-07**
rs6816491		4	69702631	9.63E-07	3.98E-01	5.88E-02	**3.14E-03**
rs7770848		6	44769237	9.63E-07	1.57E-01	1.03E-01	**2.09E-07**
rs2537798		12	10417260	9.63E-07	6.12E-01	8.33E-02	**1.39E-04**
rs6721181		2	40115696	1.24E-06	**2.54E-02**	7.06E-01	**6.05E-07**
rs17024537	*RBMS3*	3	29923279	1.24E-06	1.00E+00	4.14E-01	**1.23E-04**
rs571525		3	118162866	1.24E-06	NA	NA	NA
rs13110640		4	189471705	1.24E-06	4.39E-01	5.88E-02	**6.79E-05**
rs11833585	*SYT1*	12	79394379	1.29E-06	8.66E-01	1.27E-01	**1.60E-04**
rs4940107		18	49142762	1.39E-06	4.80E-01	7.06E-01	**6.65E-05**
rs991941		3	26268068	1.50E-06	**2.54E-02**	1.80E-01	**1.56E-04**
rs2023590		14	48151433	1.50E-06	**4.55E-02**	8.33E-02	**3.86E-05**
rs1188866	*REPS1*	6	139227575	1.52E-06	6.80E-01	4.84E-01	**2.64E-05**
rs11141597		9	89527458	1.53E-06	8.33E-02	1.00E+00	**2.03E-06**
rs9477834		6	18713337	1.57E-06	1.57E-01	1.00E+00	**2.76E-03**
rs17069881		13	79017249	1.57E-06	7.39E-01	NA	NA
rs11164749		1	103703923	1.62E-06	NA	NA	NA
rs325596		4	165384019	1.62E-06	5.78E-01	5.64E-01	**2.72E-04**
rs2722324	*SFRP4*	7	37948419	1.62E-06	8.08E-01	1.80E-01	**1.94E-04**
rs886448		7	24240165	1.62E-06	8.33E-02	8.33E-02	**7.24E-08**
rs1188883	*REPS1*	6	139260691	2.01E-06	5.32E-01	7.77E-01	**9.74E-06**

rs13246423		7	19420846	2.07E-06	1.00E+00	3.17E-01	**1.54E-05**
rs12256404	*SFMBT2*	10	7287771	2.10E-06	NA	3.17E-01	NA
rs11840504		13	63357351	2.28E-06	5.32E-01	5.64E-01	**1.28E-05**
rs10916563		1	229985395	2.52E-06	3.17E-01	NA	NA
rs6900407		6	17238649	2.52E-06	7.06E-01	1.03E-01	**1.08E-04**
rs7807274	*MKLN1*	7	131021099	2.52E-06	6.06E-02	1.00E+00	**3.82E-06**
rs7312640	*CNTN1*	12	41190744	2.52E-06	3.71E-01	7.39E-01	**9.22E-05**
rs12326216	*DLGAP1*	18	3854905	2.52E-06	1.31E-01	**1.26E-02**	**2.26E-03**
rs6503930	*CLTC*	17	57698736	2.56E-06	1.82E-01	8.27E-01	**3.77E-04**
rs13336239	*TEKT5*	16	10722412	2.60E-06	NA	3.17E-01	NA
rs9902627		17	19370568	2.60E-06	NA	5.64E-01	NA
rs7527074	*XPR1*	1	180645441	2.73E-06	8.81E-02	6.55E-01	**9.49E-06**
rs909481		1	34888152	2.73E-06	NA	**1.43E-02**	NA
rs13419784		2	196061261	2.73E-06	7.39E-01	1.57E-01	**1.35E-05**
rs11926007		3	153653071	2.73E-06	1.06E-01	4.14E-01	**1.74E-05**
rs9883878		3	177855632	2.73E-06	**2.91E-02**	1.00E+00	**1.29E-06**
rs17218161		4	59213845	2.73E-06	1.57E-01	**4.68E-03**	**1.54E-08**
rs17133682	*C7ORF72*	7	50142953	2.73E-06	4.80E-01	1.80E-01	**1.01E-04**
rs10093753	*TRIM55*	8	67076593	2.73E-06	4.67E-01	5.64E-01	**6.02E-05**
rs4242987		13	56070736	2.73E-06	NA	1.80E-01	NA
rs16952865		18	44969842	2.73E-06	4.33E-01	4.80E-01	**7.53E-03**
rs7513668		1	222295588	2.98E-06	5.32E-01	1.00E+00	**1.94E-04**
rs13361976		5	84196112	3.44E-06	8.27E-01	1.00E+00	**3.12E-04**
rs11072541	*RYR3*	15	33847380	3.44E-06	5.27E-01	3.17E-01	**9.76E-06**
rs9664323	*CUBN*	10	17094675	3.67E-06	4.05E-01	7.63E-01	**2.85E-05**
rs9297216		8	34045261	3.82E-06	**3.96E-02**	8.35E-01	**1.01E-06**
rs9483154		6	130945122	3.90E-06	NA	NA	NA
rs7563611		2	74337039	4.23E-06	1.97E-01	**4.55E-02**	**9.11E-04**
rs9823506	*ABI3BP*	3	100476713	4.23E-06	**1.14E-02**	1.57E-01	**5.79E-08**
rs41330046	*CSMD1*	8	3787060	4.23E-06	3.71E-01	**2.54E-02**	**6.89E-04**
rs16913811	*C9ORF5*	9	111861345	4.23E-06	1.34E-01	**4.55E-02**	**1.75E-03**
rs16958402	*LCN*	13	101770876	4.23E-06	5.49E-01	8.33E-02	**6.79E-05**
rs17005379		1	219564331	4.59E-06	NA	**1.43E-02**	NA
rs17105619		1	81352846	4.59E-06	1.34E-01	1.00E+00	**1.54E-05**
rs17037715		4	108387921	4.59E-06	7.15E-01	1.80E-01	**5.12E-04**
rs35141484	*KLHL5*	4	39088341	4.59E-06	2.57E-01	**1.43E-02**	**2.68E-07**
rs6816195		4	90049222	4.59E-06	6.95E-01	**4.68E-03**	**2.72E-04**
rs16892337		5	63139240	4.59E-06	9.56E-02	7.06E-01	**1.77E-04**
rs9443694		6	80451365	4.59E-06	1.32E-01	**4.55E-02**	**8.58E-04**
rs17170211	*BBS9*	7	33419486	4.59E-06	7.82E-01	3.17E-01	**3.86E-04**
rs9300554		13	88236464	4.59E-06	2.48E-01	NA	NA
rs17112657		14	27854927	4.59E-06	4.91E-01	**4.55E-02**	**2.43E-05**
rs690101		15	53402646	4.59E-06	4.39E-01	1.57E-01	**1.18E-03**

rs1527119	*SYT1*	12	79440014	4.98E-06	6.83E-01	2.01E-01	**1.64E-04**
rs1212985	*REPS1*	6	139227530	5.25E-06	4.33E-01	3.36E-01	**5.51E-05**
rs12059584	*CHRM3*	1	239905189	5.57E-06	2.48E-01	8.33E-02	**1.39E-04**
rs7217884	*STAT5A*	17	40447500	5.57E-06	7.58E-01	1.66E-01	**2.84E-04**
rs10963170	*SH3GL2*	9	17636337	5.93E-06	NA	NA	NA
rs7798651	*GT3*	7	80092919	6.13E-06	8.47E-01	**8.15E-03**	**1.47E-05**
rs7174702		15	23764447	6.25E-06	4.46E-01	1.00E+00	**1.83E-05**
rs8073039		17	57521208	6.65E-06	8.51E-02	3.43E-01	**2.24E-04**
rs6826929		4	32239198	6.79E-06	6.95E-01	7.06E-01	**4.18E-04**
rs11164524		1	102949407	7.10E-06	1.28E-01	1.57E-01	**1.90E-05**
rs16863100		2	176100049	7.10E-06	**4.12E-02**	**1.43E-02**	**1.43E-02**
rs6925916		6	132291895	7.10E-06	4.14E-01	**4.55E-02**	**3.18E-05**
rs9301462	*RAB20*	13	111207865	7.10E-06	8.82E-01	**2.70E-03**	**2.91E-04**
rs17110658		14	40906076	7.10E-06	1.00E+00	5.93E-01	**1.57E-03**
rs7231244		18	56307666	7.10E-06	3.46E-01	**8.15E-03**	**3.55E-04**
rs1628500		1	95052961	7.12E-06	NA	NA	NA
rs12270017		11	4585036	7.42E-06	6.37E-01	5.64E-01	**4.10E-04**
rs4484888		1	80652389	7.74E-06	1.80E-01	3.17E-01	**2.43E-05**

Table S2: Top 100 SNPs of African-American population. SNPs are ordered by p-values in the African American population (P2). When available, their p-values in European Americans (P1), Hispanic Americans (P3), and the mega-analysis (P123) are provided. P-values in bold are < 0.05.

rsID	Gene	Chr	BP	P2	P1	P3	P123
rs1859699		7	41065647	9.76E-06	7.01E-01	7.68E-01	8.81E-02
rs11259166	*FAM107B*	10	14580332	1.19E-05	2.10E-01	5.13E-01	**2.28E-02**
rs11259167	*FAM107B*	10	14580439	1.19E-05	3.32E-01	5.13E-01	**4.38E-02**
rs6981891		8	55391896	1.95E-05	2.27E-01	7.39E-01	**1.83E-02**
rs13004344	*LRP1B*	2	142606736	2.21E-05	6.82E-01	NA	NA
rs6463967		7	10122140	2.43E-05	**3.64E-02**	9.56E-02	8.29E-01
rs2627230		6	134379869	3.22E-05	3.55E-01	2.89E-01	9.24E-01
rs12873436		13	40868378	3.74E-05	2.00E-01	3.84E-01	8.85E-01
rs11081393		18	8524099	3.74E-05	3.23E-01	3.17E-01	7.56E-01
rs7602460		2	182261869	3.83E-05	4.77E-01	4.35E-01	**3.42E-02**
rs2221480		18	69489748	3.96E-05	1.87E-01	5.90E-01	8.85E-01
rs10811095	*FAM154A*	9	19010440	5.31E-05	1.40E-01	3.84E-01	8.79E-01
rs758240	*ENTPD5*	14	74441345	5.31E-05	1.08E-01	6.62E-01	**1.01E-02**
rs1678316		4	8336829	5.90E-05	1.00E+00	3.17E-01	**1.01E-04**
rs1145047	*CNTN4*	3	2364499	6.02E-05	2.83E-01	7.36E-02	7.93E-01
rs7926724	*TMEM135*	11	87037565	6.02E-05	3.44E-01	3.43E-01	5.55E-01
rs11621114	*ENTPD5*	14	74460095	6.02E-05	**3.02E-02**	1.00E+00	**1.82E-03**
rs12712960	*PRKCE*	2	46277923	8.87E-05	7.53E-01	7.32E-01	2.96E-01
rs1153481	*CNTN4*	3	2365123	8.87E-05	4.56E-01	**4.95E-02**	6.55E-01

rs10508477	*FAM107B*	10	14598740	9.22E-05	9.22E-01	7.73E-01	1.47E-01
rs11890111		2	130446787	9.45E-05	5.47E-01	8.08E-01	4.44E-01
rs3764859	*COQ6*	14	74425056	9.45E-05	**4.42E-02**	8.89E-01	**2.92E-03**
rs16832258	*STXBP5L*	3	121047942	9.62E-05	3.17E-01	8.33E-02	**6.71E-03**
rs10000521		4	126459422	9.62E-05	1.80E-01	NA	NA
rs12785053	*INPP5F*	10	121583965	9.64E-05	4.48E-01	5.32E-01	8.43E-02
rs10204473		2	45492633	1.01E-04	4.42E-01	3.76E-01	5.15E-01
rs1720009		4	8336465	1.01E-04	1.00E+00	3.17E-01	**2.31E-04**
rs10906697	*FAM107B*	10	14599229	1.08E-04	9.61E-01	5.64E-01	1.25E-01
rs12702976		7	10121066	1.15E-04	1.69E-01	6.70E-01	4.23E-01
rs350224	*SNX29*	16	12241873	1.15E-04	5.49E-01	1.97E-01	1.23E-01
rs340779		2	16508277	1.23E-04	8.72E-01	5.37E-01	2.99E-01
rs4766046	*TSPAN9*	12	3222714	1.24E-04	2.05E-01	4.75E-01	7.84E-02
rs12023601		1	102881189	1.26E-04	7.43E-01	8.73E-01	3.05E-01
rs11687681		2	11572815	1.26E-04	3.32E-01	1.57E-01	8.91E-01
rs555210		11	95096136	1.26E-04	6.96E-01	3.17E-01	2.33E-01
rs4269201		4	76283179	1.38E-04	9.57E-01	1.28E-01	1.11E-01
rs4831113		3	115308015	1.45E-04	1.43E-01	5.64E-01	7.81E-01
rs1988530		11	18990515	1.45E-04	NA	7.63E-01	NA
rs1499975		3	117802643	1.56E-04	2.41E-01	6.22E-01	9.23E-01
rs11099892	*KIAA0922*	4	154553693	1.56E-04	2.89E-01	7.15E-01	**4.62E-02**
rs11621118	*ENTPD5*	14	74460126	1.56E-04	1.31E-01	4.56E-01	**1.88E-02**
rs12095330		1	83266513	1.57E-04	1.00E+00	1.00E+00	**6.04E-04**
rs4656264		1	160427955	1.57E-04	1.00E+00	1.00E+00	**2.57E-03**
rs4656265		1	160427986	1.57E-04	1.00E+00	1.00E+00	**2.03E-03**
rs480745		1	83269970	1.57E-04	4.14E-01	1.00E+00	**2.46E-04**
rs2588524		2	145965660	1.57E-04	1.57E-01	5.64E-01	**9.41E-04**
rs476986		3	55493514	1.57E-04	1.00E+00	8.19E-01	2.73E-01
rs1853308		6	44551199	1.57E-04	1.00E+00	1.00E+00	**4.07E-04**
rs2473967		6	113479335	1.57E-04	**1.82E-03**	3.17E-01	**2.31E-06**
rs1384651		11	18987250	1.57E-04	2.25E-01	5.64E-01	**5.32E-04**
rs6669249	*ALG14*	1	95531373	1.61E-04	3.34E-01	8.73E-01	8.38E-01
rs17407964		1	95424669	1.62E-04	3.25E-01	4.39E-01	**1.58E-02**
rs13428810		2	154254260	1.62E-04	5.44E-01	**2.01E-02**	2.77E-01
rs9986776		7	53992161	1.62E-04	5.64E-01	3.17E-01	**2.84E-03**
rs725441	*MCPH1*	8	6263136	1.62E-04	3.17E-01	8.33E-02	**2.38E-05**
rs7948162		11	97931529	1.62E-04	9.19E-01	7.32E-01	3.22E-01
rs16964987	*PIEZO1*	16	88848279	1.62E-04	3.17E-01	5.64E-01	**1.32E-03**
rs12331558		4	158479475	1.75E-04	5.84E-01	3.36E-01	5.47E-02
rs4915564		1	197777147	1.83E-04	4.14E-01	4.75E-01	7.78E-02
rs1490548		3	153254260	1.83E-04	1.96E-01	2.89E-01	**2.59E-02**
rs11052629		12	33495533	1.94E-04	6.09E-01	3.98E-01	1.59E-01
rs10739194	*PTPRD*	9	9528291	2.08E-04	8.14E-01	1.00E+00	3.46E-01

rs10995946		10	66406460	2.08E-04	NA	NA	NA
rs6590392		11	129412688	2.13E-04	1.00E+00	7.39E-01	**3.35E-02**
rs9961693		18	59280655	2.13E-04	2.54E-01	3.17E-01	6.91E-01
rs11914436	*STXBP5L*	3	121123223	2.31E-04	**1.75E-03**	3.17E-01	2.54E-01
rs350302		5	62429113	2.31E-04	3.12E-01	7.52E-01	8.18E-01
rs7706809	*SRD5A1*	5	6635971	2.31E-04	7.86E-01	2.74E-01	3.05E-01
rs609887		11	102384567	2.31E-04	1.85E-01	3.30E-01	8.47E-01
rs2210449	*KLHL1*	13	70500548	2.31E-04	1.03E-01	5.49E-01	**5.33E-03**
rs16958724	*CDH13*	16	82864309	2.31E-04	1.15E-01	4.91E-01	7.44E-01
rs7763441		6	2452697	2.36E-04	7.34E-01	8.91E-01	1.64E-01
rs12701859		7	41072834	2.36E-04	5.93E-01	4.75E-01	3.81E-01
rs11170028		12	52558838	2.36E-04	8.87E-01	6.47E-01	3.88E-01
rs7978283		12	52559310	2.36E-04	9.63E-01	4.56E-01	3.94E-01
rs2074932	*ENTPD5*	14	74441684	2.36E-04	1.32E-01	5.55E-01	**1.91E-02**
rs10513425	*MBNL1*	3	152071820	2.39E-04	9.42E-01	6.37E-01	2.61E-01
rs6766293		3	95797766	2.39E-04	4.48E-01	6.12E-01	7.00E-02
rs16887640		5	17769086	2.39E-04	5.64E-01	NA	NA
rs2023910		7	21557854	2.39E-04	9.05E-01	1.57E-01	1.90E-01
rs6483631	*V2*	11	20050891	2.39E-04	1.85E-01	1.00E+00	**2.07E-02**
rs853751		1	214043781	2.46E-04	9.09E-01	3.69E-01	1.34E-01
rs1517631		2	224182549	2.46E-04	5.06E-02	5.88E-02	**4.44E-04**
rs645486		3	55492326	2.56E-04	8.66E-01	8.19E-01	4.31E-01
rs11259158	*FAM107B*	10	14574784	2.56E-04	3.42E-01	6.70E-01	6.38E-02
rs589524	*NBEA*	13	35731964	2.56E-04	1.15E-01	4.14E-01	5.93E-01
rs173088		16	66182658	2.56E-04	8.46E-01	5.32E-01	1.40E-01
rs17085106		18	69439073	2.56E-04	4.50E-01	1.00E+00	**2.75E-03**
rs8099373		18	69441644	2.56E-04	7.06E-01	6.55E-01	**4.51E-03**
rs10157718	*RYR2*	1	237256977	2.57E-04	**3.43E-02**	5.49E-01	1.59E-01
rs1470756		2	105449455	2.57E-04	7.46E-01	6.02E-01	2.36E-01
rs2550736		16	81437197	2.61E-04	7.74E-01	8.66E-01	4.66E-01
rs11706848	*SUCLG2*	3	67661012	2.66E-04	1.84E-01	4.23E-01	8.93E-01
rs2242022	*SUCLG2*	3	67664229	2.66E-04	2.83E-01	3.30E-01	6.89E-01
rs2190342		7	41049998	2.66E-04	1.00E+00	6.68E-01	2.22E-01
rs4454200		7	41067691	2.66E-04	8.85E-01	6.55E-01	1.76E-01
rs1393367		3	79906109	2.72E-04	5.56E-01	7.52E-01	1.02E-01
rs9830013		3	79902813	2.72E-04	8.44E-01	7.52E-01	1.99E-01
rs7688638		4	25067806	2.72E-04	**2.62E-02**	8.86E-01	**1.34E-03**
rs11781516	*ZMAT4*	8	40554742	2.72E-04	1.48E-01	2.28E-01	6.33E-01

Table S3: Top 100 SNPs of Hispanic population. SNPs are ordered by p-values in the Hispanic American population (P3). When available, their p-values in European Americans (P1), African Americans (P2), and the mega-analysis (P123) are provided. P-values in bold are < 0.05.

rsID	Gene	Chr	BP	P3	P1	P2	P123
rs1472216	*GRIP1*	12	66834976	4.20E-07	8.81E-01	5.64E-01	2.58E-01
rs7304118	*GRIP1*	12	66842135	1.29E-06	9.60E-01	1.44E-01	8.15E-02
rs11176183	*GRIP1*	12	66835453	2.10E-06	1.00E+00	6.62E-01	2.20E-01
rs326418		3	7895867	3.42E-06	9.56E-01	6.68E-01	1.87E-01
rs10052887		5	174183518	3.44E-06	7.39E-01	**4.23E-02**	**2.66E-02**
rs4704459		5	77210209	1.95E-05	8.70E-01	2.01E-01	1.04E-01
rs7134081	*GRIP1*	12	66858047	2.01E-05	7.31E-01	3.27E-01	3.28E-01
rs1776262		20	60516023	2.47E-05	9.55E-01	2.89E-01	2.83E-01
rs1831463	*GPC5*	13	92908890	2.48E-05	1.93E-01	1.17E-01	6.01E-01
rs6511312		19	22205349	2.48E-05	3.88E-01	1.49E-01	9.65E-01
rs7196508	*RBFOX1*	16	6898606	3.86E-05	4.32E-01	1.00E+00	6.39E-02
rs7987675	*GPC5*	13	92884370	3.94E-05	9.18E-02	2.97E-01	1.00E+00
rs2634737	*SUCLG2*	3	67608349	3.96E-05	6.98E-01	6.55E-01	5.79E-02
rs6845962		4	36789707	3.96E-05	3.72E-01	8.58E-01	5.37E-02
rs929131		22	26844769	4.46E-05	9.23E-01	5.16E-01	5.45E-02
rs7132670	*GRIP1*	12	66861112	5.70E-05	9.08E-01	4.84E-01	4.37E-01
rs11016928	*DOCK1*	10	129047571	6.02E-05	5.61E-02	5.13E-01	**1.61E-03**
rs4849907		2	121343006	6.25E-05	8.84E-01	8.69E-01	2.26E-01
rs490662		16	5454486	6.25E-05	6.37E-01	1.57E-01	2.37E-01
rs2164169	*DEFB129*	20	206402	6.25E-05	**7.36E-03**	4.56E-01	1.30E-01
rs17604918	*KCTD1*	18	24078160	6.33E-05	8.60E-01	8.81E-02	**4.36E-02**
rs204670		20	536536	7.44E-05	2.01E-01	4.80E-01	9.47E-01
rs391664		3	7891262	7.50E-05	4.96E-01	4.14E-01	7.27E-01
rs1159974		6	126090277	8.20E-05	9.25E-01	6.12E-01	3.39E-01
rs9388451		6	126090377	8.20E-05	9.62E-01	5.64E-01	2.00E-01
rs9398787		6	126090608	8.20E-05	9.24E-01	8.53E-01	2.54E-01
rs13486	*CPM*	12	69249087	8.77E-05	**3.65E-02**	**3.89E-03**	9.17E-01
rs3741599	*CPM*	12	69250481	8.77E-05	**3.27E-02**	2.01E-01	5.83E-01
rs958187	*IFI27L2*	14	94594768	8.77E-05	5.15E-01	4.14E-01	5.06E-02
rs11711367	*GRM7*	3	7077524	9.45E-05	2.88E-01	3.30E-01	6.45E-01
rs7245790	*ZNF492*	19	22320009	9.45E-05	**1.54E-02**	5.05E-01	2.04E-01
rs8103471	*ZNF492*	19	22317314	9.45E-05	**1.78E-02**	5.05E-01	2.21E-01
rs6089568		20	60533715	9.45E-05	9.60E-01	3.46E-01	2.99E-01
rs13117183		4	43156439	9.62E-05	8.44E-01	5.88E-02	5.76E-01
rs11120499	*KCNK2*	1	215298808	9.64E-05	3.57E-01	7.52E-01	**4.82E-02**
rs10209630	*STK39*	2	169012258	9.64E-05	1.00E+00	1.00E+00	1.58E-01
rs11687036	*RASGRP3*	2	33753366	9.64E-05	4.93E-01	6.22E-01	6.49E-01
rs2023068	*PARK2*	6	162664364	1.01E-04	1.78E-01	1.00E+00	1.00E+00
rs10733978		10	59576417	1.01E-04	6.50E-02	7.24E-01	4.15E-01

rs7086393		10	58250470	1.01E-04	2.05E-01	1.05E-01	7.90E-02
rs7478060		10	59607273	1.01E-04	6.69E-02	7.82E-02	2.28E-01
rs2904515	*GRIP1*	12	66863923	1.01E-04	3.12E-01	8.86E-01	5.94E-02
rs3891950	*GRIP1*	12	66863842	1.01E-04	5.75E-01	8.86E-01	1.38E-01
rs17545624		19	31340752	1.01E-04	9.57E-01	8.27E-01	2.10E-01
rs6429695	*KAZN*	1	15273115	1.26E-04	7.19E-01	8.27E-01	1.07E-01
rs11128174		3	66706426	1.26E-04	8.74E-01	7.07E-02	7.90E-02
rs9321054	*HEY2*	6	126079968	1.26E-04	7.76E-01	5.64E-01	3.12E-01
rs498768		12	63759333	1.26E-04	**3.88E-02**	5.08E-01	**1.39E-03**
rs6705911	*PPP1R1C*	2	182979076	1.38E-04	7.73E-01	2.21E-01	2.63E-01
rs225885		14	30471987	1.38E-04	6.39E-01	4.75E-01	3.27E-01
rs1793476	*GDPD4*	11	76992664	1.39E-04	5.56E-01	3.07E-01	1.47E-01
rs6751259	*SNTG2*	2	1064808	1.45E-04	2.18E-01	6.95E-01	**3.71E-02**
rs1350431	*PDZRN4*	12	41819315	1.45E-04	3.04E-01	1.05E-01	**1.69E-02**
rs6686296	*GRIK3*	1	37489380	1.48E-04	9.19E-01	4.23E-01	4.31E-01
rs4778020		15	92726702	1.48E-04	3.29E-01	4.92E-01	8.51E-02
rs10404620	*ZNF492*	19	22316577	1.48E-04	**1.35E-02**	2.37E-01	1.34E-01
rs6061921		20	60533512	1.48E-04	4.13E-01	1.44E-01	1.00E+00
rs4424497		1	238629579	1.56E-04	6.90E-01	3.62E-01	7.20E-01
rs2634743	*SUCLG2*	3	67612285	1.56E-04	5.30E-01	6.55E-01	7.31E-02
rs2772447	*SUCLG2*	3	67613201	1.56E-04	5.79E-01	5.47E-01	9.33E-02
rs6486070	*TEAD1*	11	12932979	1.56E-04	2.31E-01	1.70E-01	**4.26E-03**
rs2121798		3	74654727	1.57E-04	1.83E-01	6.80E-01	**2.65E-02**
rs3747134		22	26877981	1.57E-04	9.36E-01	7.73E-01	1.32E-01
rs739291	*HPS4*	22	26877539	1.57E-04	8.11E-01	7.73E-01	1.02E-01
rs6679536	*DPYD*	1	97821302	1.61E-04	6.56E-02	**4.74E-02**	**5.78E-04**
rs10405541		19	31343577	1.61E-04	8.27E-01	8.66E-01	2.14E-01
rs10405722		19	31343593	1.61E-04	9.13E-01	7.15E-01	2.70E-01
rs11085515		19	22426520	1.61E-04	**1.37E-02**	8.96E-01	2.05E-01
rs17628232		19	31342269	1.61E-04	9.13E-01	1.00E+00	2.27E-01
rs214341	*CAPZB*	1	19680558	1.62E-04	7.75E-01	4.75E-01	3.35E-01
rs6548183	*SNTG2*	2	1076968	1.62E-04	1.94E-01	**4.23E-02**	**2.58E-03**
rs1472476	*GRM7*	3	7052542	1.62E-04	3.92E-01	2.37E-01	5.01E-01
rs871262		10	59578395	1.62E-04	8.04E-02	7.39E-01	4.43E-01
rs9804334		10	57850645	1.62E-04	7.00E-01	6.55E-01	2.49E-01
rs11871636	*RPL27*	17	41154817	1.62E-04	5.02E-01	1.00E+00	8.81E-02
rs11085514		19	22425837	1.62E-04	**3.53E-02**	3.96E-01	2.17E-01
rs17118414		1	59392781	1.83E-04	3.05E-01	5.64E-01	**3.67E-02**
rs2352934		16	86187627	1.94E-04	3.37E-01	2.85E-01	1.05E-01
rs5997093	*HPS4*	22	26851828	2.08E-04	7.65E-01	8.66E-01	9.37E-02
rs4568144		3	83952437	2.13E-04	2.45E-01	7.52E-01	**4.13E-02**
rs1981933		4	76034791	2.13E-04	5.35E-01	3.17E-01	4.77E-01
rs4771988	*MBNL2*	13	97911563	2.13E-04	9.15E-01	8.66E-01	2.65E-01

rs17136317	*ZNF75A*	16	3366513	2.13E-04	7.80E-01	8.82E-01	1.65E-01
rs1978718	*ZNF492*	19	22293126	2.31E-04	**7.96E-03**	7.52E-01	1.37E-01
rs876426		2	72153022	2.39E-04	2.81E-01	8.82E-01	**3.69E-02**
rs1481244		4	67839069	2.39E-04	2.23E-01	8.64E-02	4.90E-01
rs4384322		10	57778783	2.39E-04	6.69E-01	3.04E-01	3.48E-01
rs4782023	*XYLT1*	16	17260044	2.46E-04	1.00E+00	1.00E+00	2.19E-01
rs2079557	*ARHGAP44*	17	12726712	2.46E-04	5.37E-01	8.86E-01	5.55E-01
rs10805927		5	78843271	2.56E-04	2.62E-01	8.64E-02	1.31E-01
rs4717436		7	67618930	2.56E-04	2.42E-01	**3.43E-02**	1.32E-01
rs10138605		14	101135965	2.56E-04	8.82E-01	5.05E-01	1.24E-01
rs13332693		16	75957806	2.56E-04	2.68E-01	8.66E-01	**4.62E-02**
rs323496	*RUNDC1*	17	41141971	2.56E-04	2.96E-01	6.02E-01	6.72E-02
rs739289	*HPS4*	22	26862153	2.56E-04	9.36E-01	4.56E-01	1.27E-01
rs4878088	*DAPK1*	9	90141193	2.57E-04	1.85E-01	1.80E-01	1.00E+00
rs4576739		10	58261408	2.57E-04	4.03E-01	1.14E-01	2.04E-01
rs4483759		13	45676009	2.57E-04	8.97E-01	1.70E-01	7.18E-02
rs9567523		13	45660986	2.57E-04	1.00E+00	1.23E-01	8.26E-02
rs12977165		19	31345459	2.57E-04	1.00E+00	8.47E-01	3.10E-01

Table S4: Top 100 SNPs of the mega-analysis. SNPs are ordered by p-values in the mega-analysis (P123). When available, their p-values in European Americans (P1), African Americans (P2), and Hispanic Americans (P3) are provided. P-values in bold are < 0.05.

rsID	Gene	chr	BP	P123	P1	P2	P3
rs16929097		9	12521826	8.33E-09	**1.81E-07**	**1.14E-02**	4.14E-01
rs17218161		4	59213845	1.54E-08	**2.73E-06**	1.57E-01	**4.68E-03**
rs12570188	*HPSE2*	10	100855702	4.60E-08	**3.19E-07**	8.33E-02	1.57E-01
rs9823506	*ABI3BP*	3	100476713	5.79E-08	**4.23E-06**	**1.14E-02**	1.57E-01
rs920672	*V2*	11	20050143	5.79E-08	**2.67E-05**	**1.43E-02**	**1.43E-02**
rs886448		7	24240165	7.24E-08	**1.62E-06**	8.33E-02	8.33E-02
rs11000019	*PSAP*	10	73591530	7.62E-08	**4.46E-07**	3.17E-01	8.33E-02
rs6054973		20	7385958	1.11E-07	**5.34E-07**	1.66E-01	**4.55E-02**
rs2292527	*FLCN*	17	17126041	1.50E-07	**8.81E-06**	**1.14E-02**	1.32E-01
rs16925517		9	109247001	1.90E-07	**3.38E-05**	**4.55E-02**	**1.43E-02**
rs7974135		12	108371313	1.90E-07	**1.31E-05**	**3.39E-02**	**4.55E-02**
rs7770848		6	44769237	2.09E-07	**9.63E-07**	1.57E-01	1.03E-01
rs1530976	*SLC4A4*	4	72393391	2.68E-07	**7.74E-06**	**6.66E-03**	5.64E-01
rs35141484	*KLHL5*	4	39088341	2.68E-07	**4.59E-06**	2.57E-01	**1.43E-02**
rs928382		10	121881681	3.19E-07	**2.21E-05**	1.03E-01	**1.43E-02**
rs4695314		4	47932661	3.56E-07	**7.74E-06**	**4.95E-02**	**1.96E-02**
rs17033506		3	35639826	4.46E-07	**9.63E-07**	4.14E-01	8.33E-02
rs17111521		14	41633385	4.84E-07	**2.70E-03**	**4.18E-04**	**2.54E-02**
rs7045156		9	81926265	5.73E-07	**3.74E-05**	**2.50E-03**	3.17E-01

rs6721181		2	40115696	6.05E-07	**1.24E-06**	**2.54E-02**	7.06E-01
rs1314595	*ATRNL1*	10	116908479	6.91E-07	**3.12E-04**	**1.16E-02**	**8.15E-03**
rs11021111		11	95003263	7.98E-07	**5.56E-04**	**1.16E-02**	**1.26E-02**
rs2928442		10	59146466	8.68E-07	**1.39E-04**	1.17E-01	**1.62E-03**
rs3814399	*BOC*	3	112991492	8.95E-07	**3.38E-05**	1.57E-01	**2.54E-02**
rs9590225	*ABCC4*	13	95916201	8.95E-07	**2.21E-05**	5.88E-02	8.33E-02
rs16882243	*SEMA5A*	5	9226440	9.49E-07	**1.08E-04**	**2.09E-02**	**3.39E-02**
rs9297216		8	34045261	1.01E-06	**3.82E-06**	**3.96E-02**	8.35E-01
rs7613331		3	1533549	1.14E-06	**1.95E-05**	5.88E-02	1.57E-01
rs35216186		3	83038704	1.24E-06	**1.60E-05**	1.57E-01	8.33E-02
rs9883878		3	177855632	1.29E-06	**2.73E-06**	**2.91E-02**	1.00E+00
rs41479544	*ATP8B1*	18	55472316	1.29E-06	**1.35E-05**	5.64E-01	**2.54E-02**
rs2218416		18	65360863	1.38E-06	**1.04E-04**	**6.71E-03**	2.48E-01
rs7561701		2	138472227	1.48E-06	**2.01E-05**	1.08E-01	**1.43E-02**
rs17129414	*PDZRN4*	12	41893994	1.57E-06	**9.58E-06**	1.03E-01	3.17E-01
rs943806	*FAM102B*	1	109156284	1.62E-06	**3.12E-04**	**8.15E-03**	8.33E-02
rs2705520	*ATG3*	3	112269287	1.87E-06	**8.95E-07**	5.64E-01	1.03E-01
rs11141597		9	89527458	2.03E-06	**1.53E-06**	8.33E-02	1.00E+00
rs2451167		8	119152919	2.07E-06	**1.08E-04**	8.33E-02	**2.09E-02**
rs2472669	*NR1I2*	3	119501960	2.17E-06	**1.47E-05**	5.94E-02	2.57E-01
rs2473967		6	113479335	2.31E-06	**1.82E-03**	**1.57E-04**	3.17E-01
rs674467		11	104812647	2.38E-06	**3.89E-03**	**1.02E-03**	**4.55E-02**
rs6733115		2	217488954	2.43E-06	**7.74E-06**	1.03E-01	**2.54E-02**
rs2272266	*PLA1A*	3	119337198	2.43E-06	**1.23E-04**	6.55E-01	**1.57E-03**
rs1418489	*CAMTA1*	1	7425064	2.52E-06	**6.33E-05**	5.88E-02	8.33E-02
rs17083580	*KIAA0825*	5	93648131	2.52E-06	**1.31E-05**	3.17E-01	8.33E-02
rs2159424		5	126487366	2.52E-06	**1.08E-04**	**1.96E-02**	1.57E-01
rs1295378		6	119685952	2.52E-06	**2.21E-05**	5.64E-01	**2.54E-02**
rs17152730		10	126911566	2.52E-06	**1.31E-05**	3.17E-01	1.03E-01
rs12168899		22	50101815	2.60E-06	**6.33E-05**	**3.25E-02**	8.33E-02
rs1415649		9	104636695	2.69E-06	**1.48E-05**	4.14E-01	9.56E-02
rs6600391	*HIVEP3*	1	42371447	2.73E-06	**3.12E-04**	**4.55E-02**	**2.54E-02**
rs7006993		8	83889759	3.06E-06	**7.44E-05**	5.78E-02	8.33E-02
rs12359404	*SORCS1*	10	108334189	3.10E-06	**4.07E-04**	**5.32E-04**	3.17E-01
rs2807510	*RIMS1*	6	73090537	3.42E-06	**1.95E-05**	1.80E-01	1.80E-01
rs11007764		10	30116104	3.44E-06	**3.47E-04**	**4.55E-02**	**2.54E-02**
rs10400286	*KCNQ1*	11	2616053	3.44E-06	**1.31E-05**	1.57E-01	1.57E-01
rs17010574	*GALNT14*	2	31243964	3.67E-06	**2.67E-05**	3.66E-01	**1.43E-02**
rs6882632		5	159017398	3.67E-06	**1.19E-05**	1.34E-01	8.33E-02
rs872101		3	67298578	3.71E-06	**6.33E-05**	**2.36E-02**	**2.54E-02**
rs10005234		4	68319533	3.82E-06	**3.61E-05**	5.94E-02	1.57E-01
rs7807274	*MKLN1*	7	131021099	3.82E-06	**2.52E-06**	6.06E-02	1.00E+00
rs9918659		7	54039000	3.82E-06	**3.38E-05**	**3.90E-02**	1.32E-01

rs2303409	*PGM2*	4	37846939	4.23E-06	**6.33E-05**	3.17E-01	**3.39E-02**
rs16944951		16	51820998	4.23E-06	**1.57E-03**	**1.14E-02**	**2.54E-02**
rs2031662		13	41610561	4.30E-06	**1.19E-05**	2.57E-01	1.57E-01
rs7908624		10	132509961	4.47E-06	**5.31E-05**	2.85E-01	**3.25E-02**
rs16976458		17	69196263	4.59E-06	**2.21E-05**	3.17E-01	1.57E-01
rs17716091		17	65680034	5.00E-06	**3.61E-05**	1.80E-01	1.57E-01
rs16922480	*KANK1*	9	678010	5.02E-06	**2.01E-05**	7.06E-01	**1.43E-02**
rs17036023	*IGSF3*	1	117129711	5.20E-06	**2.04E-07**	6.95E-01	**1.43E-02**
rs2155668		18	13799456	5.58E-06	**1.39E-04**	3.17E-01	**8.15E-03**
rs1439706		2	35764414	5.75E-06	**2.01E-05**	1.00E+00	**4.55E-02**
rs733187		2	175602377	5.75E-06	**5.32E-04**	**2.09E-02**	**4.55E-02**
rs16888111	*WDR27*	6	169935826	5.75E-06	**5.70E-05**	**2.54E-02**	5.64E-01
rs9925521	*CDH13*	16	83457455	5.75E-06	**2.01E-05**	5.64E-01	8.33E-02
rs7552300	*NFIA*	1	61688282	5.82E-06	**1.95E-05**	1.66E-01	1.32E-01
rs1327013		10	130573275	5.90E-06	**3.80E-04**	**7.53E-03**	1.03E-01
rs12070405		1	43357369	5.93E-06	**1.31E-05**	2.85E-01	**3.39E-02**
rs1036966		8	126789900	5.93E-06	**1.34E-03**	**1.05E-02**	**2.54E-02**
rs16990878	*EFCAB6*	22	44042348	5.93E-06	**7.89E-04**	**7.29E-03**	1.03E-01
rs10128616	*GRM5*	11	88286437	6.03E-06	**2.75E-04**	**1.58E-02**	**4.55E-02**
rs13374752	*TMEM61*	1	55453391	6.11E-06	**3.74E-05**	8.81E-02	**2.54E-02**
rs7716850		5	87430517	6.11E-06	**2.56E-04**	9.56E-02	**3.39E-02**
rs4692508		4	31354090	6.36E-06	**1.88E-05**	1.49E-01	5.78E-01
rs1935521	*KCNQ5*	6	73573771	7.10E-06	**3.89E-03**	**2.70E-03**	8.33E-02
rs3849488	*ROBO2*	3	76464685	7.74E-06	**1.83E-04**	8.33E-02	8.33E-02
rs9836097		3	74591012	7.74E-06	**2.34E-04**	**4.55E-02**	**2.54E-02**
rs12280132	*HRASLS5*	11	63231259	7.74E-06	**3.12E-04**	**1.43E-02**	3.17E-01
rs7157695	*WDR25*	14	100896800	7.74E-06	**3.12E-04**	1.57E-01	**2.54E-02**
rs17837682		7	156352856	8.24E-06	**5.31E-05**	8.33E-02	3.17E-01
rs41334648		13	23200009	8.24E-06	**1.69E-05**	1.00E+00	7.07E-02
rs11877823		18	57662696	8.42E-06	**1.01E-04**	2.39E-01	**6.66E-03**
rs10219720		12	79109021	9.10E-06	**1.57E-03**	**1.18E-03**	3.17E-01
rs12607175	*DLGAP1*	18	4147483	9.10E-06	**1.08E-04**	**8.15E-03**	3.17E-01
rs7527074	*XPR1*	1	180645441	9.49E-06	**2.73E-06**	8.81E-02	6.55E-01
rs7835585	*TSRE1*	8	143298768	9.49E-06	**5.32E-04**	**8.15E-03**	9.56E-02
rs10059123		5	154442093	9.55E-06	**1.83E-04**	**3.90E-02**	5.88E-02
rs10120589		9	7391855	9.55E-06	**2.01E-05**	2.48E-01	1.03E-01
rs1981993	*CD82*	11	44607471	9.55E-06	**1.60E-03**	**4.55E-02**	**8.15E-03**
rs11940540		4	36066470	9.58E-06	**6.36E-04**	**1.43E-02**	9.56E-02

Table S5: Number of SNPs with p-value below cutoffs.

					Overlap			
**p-value cutoff**	EA	AA	HA	Mega	EA&AA	EA&HA	AA&HA	EA, AA&HA
**0.05**	58857	39658	39618	51638	2722	3270	1962	180
**0.01**	15493	6538	6557	13250	97	132	48	2
**1×10^{-4}**	632	25	37	462	0	0	0	0
**1×10^{-5}**	126	1	5	106	0	0	0	0
**1×10^{-6}**	22	0	1	26	0	0	0	0

EA= European American, AA= African American, HA= Hispanic American populations, Mega: Mega-analysis based on combined samples from all three populations.

Table S6: top 1% SNPs of RYR2 after imputation for each population.

			P-value			
rsID	BP	EA	AA	HA	Region	Possible Functional Effects
European Americans						
**rs16835325**	237659306	2.55E-07	0.162	0.763	intronic	protein coding
**rs2797436**	237863718	0.000789	0.0105	0.317	Synonymous coding	protein coding
rs12136903	237966877	0.000789	0.157	1	downstream, intronic	processed transcript, protein coding
**rs3820216**	237777828	0.000911	1	0.414	Synonymous coding	Protein coding
rs2253083	237929556	0.00365	0.491	0.655	intronic	protein coding
**rs1953076**	237826332	0.00468	0.223	0.317	intronic	protein coding
NA	237929787	0.00491	0.491	0.655		
rs12737847	237945870	0.00596	0.0164	0.655	intronic	protein coding
NA	237946481	0.00596	0.00729	0.655		
NA	237929790	0.00658	0.0896	0.317		
rs113408406	237934206	0.00666	NA	0.317	intronic	protein coding
rs10925507	237913281	0.0071	0.394	1	intronic	protein coding
rs12755289	237942500	0.00763	0.0719	0.655	intronic	protein coding
NA	237773235	0.00804	NA	0.763		
rs80013027	237941945	0.0103	0.0833	1	intronic	protein coding
**rs2248295**	237729774	0.0126	1	0.593	intronic	protein coding
**rs6682119**	237618227	0.0143	0.0956	0.564	intronic	protein coding
rs140318912	237830680	0.0143	NA	NA	intronic	protein coding
rs114813259	238015916	0.0143	NA	NA	intronic	protein coding
rs145687313	237939429	0.0164	0.317	1	intronic	protein coding
rs79387075	237939590	0.0164	1	1	intronic	protein coding
rs143222085	237940597	0.0164	0.317	1	intronic	protein coding
rs148063848	237940611	0.0164	0.317	1	intronic	protein coding
rs143686364	237940973	0.0164	0.317	1	intronic	protein coding
rs147193397	237941144	0.0164	0.317	1	intronic	protein coding

**African Americans**						
rs2685301	237890437	NA	0.000415	0.317	synonymous coding, 3prime utr	protein coding
**rs2797447**	237905342	0.0254	0.00109	1	intronic	protein coding
rs2819766	237917047	0.317	0.00109	0.103	intronic	protein coding
rs2685289	237909539	0.317	0.0017	0.48	intronic	protein coding
**rs2797441**	237881770	NA	0.0025	1	3prime utr, synonymous coding	protein coding
**rs6429032**	237805925	0.936	0.00389	0.405	intronic	protein coding
rs2177065	237915007	0.0278	0.00406	1	intronic	protein coding
rs3766877	237808779	0.744	0.00443	0.763	intronic	protein coding
rs1879673	237895805	NA	0.00607	0.317	intronic	protein coding
rs141708048	237815112	NA	0.00666	0.317	intronic	protein coding
rs2819769	237911369	NA	0.00706	0.564	intronic	protein coding
NA	237946481	0.00596	0.00729	0.655		
rs6669057	237638942	1	0.00793	0.527	intronic	protein coding
NA	237741600	0.286	0.00793	0.0412		
rs11583033	237809064	0.744	0.00815	1	intronic	protein coding
rs1987939	237751942	0.96	0.00944	0.384	intronic	protein coding
**rs2685296**	237898423	NA	0.00944	0.655	intronic	protein coding
**rs2797436**	237863718	0.000789	0.0105	0.317	synonymous coding	protein coding
rs939699	237865203	NA	0.0105	1	intronic	protein coding
**rs2779431**	237755348	0.157	0.0124	0.317	intronic	protein coding
rs12136895	237757370	0.954	0.0133	0.0105	intronic	protein coding
rs10925429	237637687	1	0.0136	0.527	intronic	protein coding
rs10925430	237637880	1	0.0136	0.527	intronic	protein coding
rs10925431	237637930	1	0.0136	0.527	intronic	protein coding
rs10754607	237638053	1	0.0136	0.527	intronic	protein coding
rs10754608	237638182	1	0.0136	0.527	intronic	protein coding
NA	237639225	0.919	0.0136	0.527		
rs6429019	237639394	0.798	0.0136	0.527	intronic	protein coding
rs6663913	237639870	0.798	0.0136	0.527	intronic	protein coding
**Hispanic Americans**						
**rs34729156**	237735016	0.738	0.842	0.00118	intronic	protein coding
rs2779395	237726963	0.323	0.18	0.0016	intronic	protein coding
NA	237727031	0.408	0.18	0.0016		
rs2392628	237745795	0.957	0.139	0.003	intronic	protein coding
rs4415553	237745782	0.914	0.317	0.00319	intronic	protein coding
rs2805475	237726500	0.439	0.16	0.00349	intronic	protein coding
rs2805477	237726909	0.408	0.18	0.00349	intronic	protein coding
rs2779396	237727085	0.409	0.14	0.00349	intronic	protein coding
rs722581	237725727	0.781	0.835	0.0035	intronic	protein coding
rs7540869	237598400	0.317	0.547	0.00389	intronic	protein coding

NA	237598877	0.317	0.547	0.00389		
rs2805388	237727057	0.408	0.14	0.00389	intronic	protein coding
rs61832624	237737107	0.78	0.842	0.00443	intronic	protein coding
rs6689392	237737862	0.824	0.842	0.00443	intronic	protein coding
rs61832625	237741696	0.781	0.847	0.00443	intronic	protein coding
rs2127157	237747986	0.734	0.405	0.00443	intronic	protein coding
rs61832626	237748340	0.822	0.405	0.00443	intronic	protein coding
NA	237758424	0.908	0.593	0.00443		
rs2127159	237762445	0.77	0.593	0.00443		
**rs2779397**	237727112	0.445	0.14	0.00468		
rs2779393	237725353	0.345	0.128	0.00535		
rs2618716	237676668	0.692	0.706	0.00607		
rs61832596	237723742	0.766	0.45	0.00649		

Bolded SNPs were genotyped in our study data, others were imputed.

Table S7: Top 30 pathways of each population when top 1,000 SNPs are declared as noteworthy. The order of the pathways corresponds to their significant level. Pathways in bold are shared by two of the populations. There is no pathway shared by all the three populations.

European American (p-values: 0.0008 – 0.0762)	***P-value***
BIOCARTA G2 PATHWAY	0.0008
REACTOME E2F TRANSCRIPTIONAL TARGETS AT G1 S	0.0034
REACTOME E2F MEDIATED REGULATION OF DNA REPLICATION	0.0036
BIOCARTA PTC1 PATHWAY	0.0138
SA G2 AND M PHASES	0.0168
REACTOME G2 M CHECKPOINTS	0.0178
REACTOME E2F ENABLED INHIBITION OF PRE REPLICATION COMPLEX FORMATION	0.0198
**BIOCARTA STATHMIN PATHWAY**	**0.0204**
BIOCARTA ATM PATHWAY	0.0268
REACTOME CYCLIN A1 ASSOCIATED EVENTS DURING G2 M TRANSITION	0.032
REACTOME CELL CELL ADHESION SYSTEMS	0.0376
REACTOME REMOVAL OF THE FLAP INTERMEDIATE	0.038
BIOCARTA G1 PATHWAY	0.044
REACTOME CREB PHOSPHORYLATION THROUGH THE ACTIVATION OF CAMKII	0.0448
REACTOME RNA POLYMERASE III TRANSCRIPTION INITIATION FROM TYPE 2 PROMOTER	0.0482
KEGG CELL CYCLE	0.0484
BIOCARTA P35ALZHEIMERS PATHWAY	0.0494
REACTOME ACTIVATION OF ATR IN RESPONSE TO REPLICATION STRESS	0.0506
BIOCARTA PITX2 PATHWAY	0.0552
BIOCARTA EIF2 PATHWAY	0.0554
BIOCARTA PTDINS PATHWAY	0.0584

KEGG NON HOMOLOGOUS END JOINING	0.0594
REACTOME LAGGING STRAND SYNTHESIS	0.0628
KEGG N GLYCAN BIOSYNTHESIS	0.063
REACTOME RETROGRADE NEUROTROPHIN SIGNALLING	0.0636
BIOCARTA CACAM PATHWAY	0.0682
REACTOME RNA POLYMERASE III TRANSCRIPTION	0.0714
REACTOME AMINE COMPOUND SLC TRANSPORTERS	0.0718
BIOCARTA SRCRPTP PATHWAY	0.0742
REACTOME RAS ACTIVATION UOPN CA2 INFUX THROUGH NMDA RECEPTOR	0.0762
African American (p-values: 0.0032 – 0.0866)	
KEGG ALANINE ASPARTATE AND GLUTAMATE METABOLISM	0.0032
BIOCARTA LONGEVITY PATHWAY	0.0088
KEGG MELANOMA	0.0162
REACTOME EFFECTS OF PIP2 HYDROLYSIS	0.0204
REACTOME SIGNALING BY BMP	0.0268
BIOCARTA BAD PATHWAY	0.0306
KEGG REGULATION OF ACTIN CYTOSKELETON	0.0344
BIOCARTA P53 PATHWAY	0.0408
REACTOME MYOGENESSIS	0.0484
KEGG DILATED CARDIOMYOPATHY	0.0522
**REACTOME PREFOLDIN MEDIATED TRANSFER OF SUBSTRATE TO CCT TRIC**	**0.0532**
REACTOME ELECTRON TRANSPORT CHAIN	0.0536
REACTOME CHAPERONIN MEDIATED PROTEIN FOLDING	0.0558
KEGG PROSTATE CANCER	0.0566
BIOCARTA CTLA4 PATHWAY	0.0586
KEGG TGF BETA SIGNALING PATHWAY	0.062
KEGG HYPERTROPHIC CARDIOMYOPATHY HCM	0.0632
KEGG RENIN ANGIOTENSIN SYSTEM	0.0654
REACTOME SHCMEDIATED CASCADE	0.0662
KEGG GLYCOSAMINOGLYCAN BIOSYNTHESIS KERATAN SULFATE	0.067
BIOCARTA HDAC PATHWAY	0.069
KEGG PANCREATIC CANCER	0.0718
REACTOME PI3K CASCADE	0.0718
BIOCARTA RACCYCD PATHWAY	0.0764
BIOCARTA AKT PATHWAY	0.0768
KEGG COLORECTAL CANCER	0.078
**REACTOME CLASS C3 METABOTROPIC GLUTAMATE PHEROMONE RECEPTORS**	**0.083**
REACTOME PHOSPHOLIPASE CMEDIATED CASCADE	0.0838
KEGG SYSTEMIC LUPUS ERYTHEMATOSUS	0.086
REACTOME METABOLISM OF LIPIDS AND LIPOPROTEINS	0.0866

Hispanic American (p-values: 0.0006 – 0.0958)	
REACTOME AMINE LIGAND BINDING RECEPTORS	0.0006
REACTOME G ALPHA S SIGNALLING EVENTS	0.006
REACTOME GLUCONEOGENESIS	0.0138
KEGG CITRATE CYCLE TCA CYCLE	0.016
KEGG SPHINGOLIPID METABOLISM	0.0166
REACTOME GPCR LIGAND BINDING	0.022
BIOCARTA CK1 PATHWAY	0.0266
REACTOME OPIOID SIGNALLING	0.0342
BIOCARTA CFTR PATHWAY	0.0352
BIOCARTA GATA3 PATHWAY	0.0364
KEGG THYROID CANCER	0.0376
KEGG PRIMARY BILE ACID BIOSYNTHESIS	0.0464
**REACTOME PREFOLDIN MEDIATED TRANSFER OF SUBSTRATE TO CCT TRIC**	**0.0474**
REACTOME METABOLISM OF PROTEINS	0.0504
REACTOME STRIATED MUSCLE CONTRACTION	0.0618
**REACTOME CLASS C3 METABOTROPIC GLUTAMATE PHEROMONE RECEPTORS**	**0.062**
REACTOME SYNTHESIS OF BILE ACIDS AND BILE SALTS	0.0668
REACTOME CITRIC ACID CYCLE	0.0678
ST G ALPHA S PATHWAY	0.069
REACTOME CLASS A1 RHODOPSIN LIKE RECEPTORS	0.0698
KEGG ENDOCYTOSIS	0.0708
REACTOME ADP SIGNALLING THROUGH P2Y PURINOCEPTOR 12	0.0722
REACTOME DARPP32 EVENTS	0.0768
REACTOME GLUCOSE METABOLISM	0.0796
**BIOCARTA STATHMIN PATHWAY**	**0.082**
KEGG ARRHYTHMOGENIC RIGHT VENTRICULAR CARDIOMYOPATHY ARVC	0.0852
REACTOME INHIBITION OF INSULIN SECRETION BY ADRENALINE NORADRENALINE	0.0894
KEGG PPAR SIGNALING PATHWAY	0.0906
BIOCARTA CSK PATHWAY	0.0906
REACTOME STEROID METABOLISM	0.0958

Table S8: Top 30 GO terms of each population when top 1K SNPs are declared as noteworthy. The order of the GOs corresponds to their significant level. GOs in bold are shared by two of the populations. There is no pathway shared by all the three populations.

European American (p-values: 0.0006 - 0.0670)	***p-value***
CELLULAR RESPONSE TO STRESS	0.0006
VITAMIN BINDING	0.0056
JAK STAT CASCADE	0.0062

VITAMIN TRANSPORT	0.0072
ER NUCLEAR SIGNALING PATHWAY	0.0078
REGULATION OF GENE SPECIFIC TRANSCRIPTION	0.0078
CELLULAR RESPONSE TO STIMULUS	0.0110
S PHASE OF MITOTIC CELL CYCLE	0.0150
TRANSCRIPTION COREPRESSOR ACTIVITY	0.0198
CALCIUM INDEPENDENT CELL CELL ADHESION	0.0204
PHOSPHORIC DIESTER HYDROLASE ACTIVITY	0.0210
LATE ENDOSOME	0.0248
S PHASE	0.0314
REGULATION OF CELL CYCLE	0.0342
TRANSCRIPTION FACTOR BINDING	0.0342
NEGATIVE REGULATION OF CELLULAR COMPONENT ORGANIZATION AND BIOGENESIS	0.0354
CONDENSED CHROMOSOME	0.0414
3 5 CYCLIC NUCLEOTIDE PHOSPHODIESTERASE ACTIVITY	0.0432
CYCLIC NUCLEOTIDE PHOSPHODIESTERASE ACTIVITY	0.0438
TRANSCRIPTION REPRESSOR ACTIVITY	0.0464
MITOCHONDRIAL MEMBRANE	0.0508
MITOCHONDRIAL MEMBRANE PART	0.0510
MEMBRANE LIPID BIOSYNTHETIC PROCESS	0.0550
RESPONSE TO DNA DAMAGE STIMULUS	0.0556
REGULATION OF HEART CONTRACTION	0.0564
**RIBONUCLEASE ACTIVITY**	**0.0576**
LIGASE ACTIVITY	0.0634
COFACTOR BINDING	0.0638
TRANSCRIPTION FACTOR COMPLEX	0.0642
DNA DAMAGE RESPONSESIGNAL TRANSDUCTION	0.0670
African American (p-values: 0.0006 - 0.0448)	
POSITIVE REGULATION OF CELL PROLIFERATION	0.0006
LYSOSOMAL TRANSPORT	0.0016
CARBOHYDRATE BINDING	0.0038
RNA CATABOLIC PROCESS	0.0050
VACUOLAR TRANSPORT	0.0052
SUGAR BINDING	0.0120
PYRIMIDINE NUCLEOTIDE METABOLIC PROCESS	0.0150
SENSORY PERCEPTION OF CHEMICAL STIMULUS	0.0172
BIOPOLYMER CATABOLIC PROCESS	0.0198
UBIQUITIN LIGASE COMPLEX	0.0220
RECEPTOR SIGNALING PROTEIN SERINE THREONINE KINASE ACTIVITY	0.0226
REGULATION OF CELL PROLIFERATION	0.0232
CELL PROLIFERATION GO 0008283	0.0242
POSITIVE REGULATION OF CELLULAR PROCESS	0.0246

LYSOSOME ORGANIZATION AND BIOGENESIS	0.0286
NUCLEAR UBIQUITIN LIGASE COMPLEX	0.0308
VACUOLE ORGANIZATION AND BIOGENESIS	0.0312
GLYCOSAMINOGLYCAN BINDING	0.0318
BILE ACID METABOLIC PROCESS	0.0326
IMMUNE EFFECTOR PROCESS	0.0338
STRUCTURAL MOLECULE ACTIVITY	0.0358
DETECTION OF CHEMICAL STIMULUS	0.0374
**MACROMOLECULE CATABOLIC PROCESS**	**0.0374**
POSITIVE REGULATION OF BIOLOGICAL PROCESS	0.0376
POLYSACCHARIDE BINDING	0.0400
**CELLULAR MACROMOLECULE CATABOLIC PROCESS**	**0.0404**
**RIBONUCLEASE ACTIVITY**	**0.0436**
GOLGI MEMBRANE	0.0444
STRUCTURAL CONSTITUENT OF CYTOSKELETON	0.0444
REGULATION OF MEMBRANE POTENTIAL	0.0448
Hispanic American (p-values: 0.0004-0.0282)	
NEGATIVE REGULATION OF CATALYTIC ACTIVITY	0.0004
PROTEIN KINASE BINDING	0.0016
KINASE BINDING	0.0040
REGULATION OF TRANSLATION	0.0050
ANION TRANSMEMBRANE TRANSPORTER ACTIVITY	0.0080
NEGATIVE REGULATION OF HYDROLASE ACTIVITY	0.0100
NEGATIVE REGULATION OF TRANSFERASE ACTIVITY	0.0104
REGULATION OF PROTEIN METABOLIC PROCESS	0.0110
CATABOLIC PROCESS	0.0124
CELLULAR BIOSYNTHETIC PROCESS	0.0152
ECTODERM DEVELOPMENT	0.0156
REGULATION OF GENE EXPRESSION	0.0168
TRANSLATION	0.0170
**MACROMOLECULE CATABOLIC PROCESS**	**0.0184**
APICAL PLASMA MEMBRANE	0.0188
BIOSYNTHETIC PROCESS	0.0196
PRODUCTION OF MOLECULAR MEDIATOR OF IMMUNE RESPONSE	0.0198
**CELLULAR MACROMOLECULE CATABOLIC PROCESS**	**0.0204**
REGULATION OF CATABOLIC PROCESS	0.0210
REGULATION OF METABOLIC PROCESS	0.0210
MACROMOLECULE BIOSYNTHETIC PROCESS	0.0224
TISSUE DEVELOPMENT	0.0242
REGULATION OF CELLULAR PROTEIN METABOLIC PROCESS	0.0246
ENZYME BINDING	0.0246
PROTEIN COMPLEX	0.0248
TRANSLATION FACTOR ACTIVITY NUCLEIC ACID BINDING	0.0248

APICAL PART OF CELL	0.0266
CYTOSKELETAL PART	0.0268
ACTIVE TRANSMEMBRANE TRANSPORTER ACTIVITY	0.0276
RESPONSE TO CARBOHYDRATE STIMULUS	0.0282
